# Mechanically Twisting‐Induced Top‐Down Chirality Transfer for Tunable Full‐Color Circularly Polarized Luminescent Fibers

**DOI:** 10.1002/advs.202412778

**Published:** 2024-12-04

**Authors:** Xiaoxiao Yu, Linfeng Chen, Qin Liu, Xiaoqing Liu, Zhenduo Qiu, Xinhai Zhang, Meifang Zhu, Yanhua Cheng

**Affiliations:** ^1^ State Key Laboratory for Modification of Chemical Fibers and Polymer Materials College of Materials Science and Engineering Donghua University Shanghai 201620 P. R. China

**Keywords:** circular polarized luminescence, fiber twisting, helical structure, top‐down chirality transfer

## Abstract

Circularly polarized luminescence (CPL) materials with rich optical information are highly attractive for optical display, information storage, and encryption. Although previous investigations have shown that external force fields can induce CPL activity in nonchiral systems, the unique role of macroscopic external forces in inducing CPL has not been demonstrated at the level of molecule or molecular aggregate. Here, a canonical example of CPL generation by mechanical twisting in an achiral system consisting of a polymer matrix with embedded fluorescent molecules is presented. By carefully adjusting the twisting parameters in time and space, in conjunction with circular dichroism (CD), CPL, and 2D wide‐angle X‐ray scattering (2D WAXS) studies, a twisting‐induced top‐down chiral transfer mechanism derived from the molecular‐level asymmetric rearrangement of fluorescent units is elucidated within polymers under external torsional forces. This top‐down chiral transfer provides a simple, scalable, and versatile mechanical twisting strategy for the fabrication of CPL materials, allowing for fabricating full‐color and handedness‐tunable CPL fibers, where the macroscopic twist direction determines the CPL handedness. Moreover, the weavability of CPL fibers greatly extend their applications in anti‐counterfeit encryption, as demonstrated by using embroidery techniques to design multilevel encryption patterns.

## Graphical abstract

A top‐down chirality transfer mechanism is presented to create macroscopic full‐color circularly polarized luminescence (CPL) fibers via the rearrangement of achiral luminophores embedded in polymers under external torsional force, where the fiber twist direction determines the handedness of CPL and the torsional density exerts influence over luminescence dissymmetry factor (*g*_lum_).

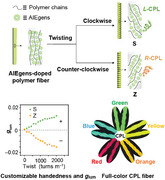

## 1 Introduction

Circularly polarized luminescence (CPL) with rich optical information has attracted significant attention across various fields including 3D optical imaging,^[^ ^1^, ^2^, ^3^, ^4^ ^]^ information encryption,^[^ ^5^, ^6^, ^7^, ^8^, ^9^ ^]^ optoelectronic devices,^[^ ^10^, ^11^, ^12^ ^]^ drug screening,^[^ ^13^, ^14^ ^]^ and asymmetric synthesis,^[^ ^15^, ^16^, ^17^, ^18^ ^]^ etc. In order to create CPL materials, scientists have devoted significant efforts to constructing chiral structures in fluorescent materials, drawing inspiration from natural chiral entities such as DNA double helix,^[^ ^19^ ^]^ peptide chains,^[^ ^20^ ^]^ and plant vines.^[^ ^21^ ^]^ Among the various chiral structures, the helical fibrous morphology is widely favored for its high luminescence dissymmetry factor (*g*_lum_) due to its higher anisotropic factor.^[^ ^22^, ^23^ ^]^ These helical fibrous morphologies are typically formed by self‐organization or self‐assembly of chiral units with *π*‐conjugated luminophores through covalent or non‐covalent interactions.^[^ ^24^, ^25^, ^26^, ^27^, ^28^ ^]^ The strategy has been termed the “bottom‐up” route, which relies heavily on the repetitive synthesis of chiral molecules and the in situ regulation of molecular chiral arrangements.^[^ ^29^ ^]^ As the scale and complexity of the required chiral assemblies increase, this approach may become unmanageable.

Distinguished from the molecular manufacturing‐based bottom‐up approach, top‐down approach (known as stepwise refinement) relying on conventional manufacturing is easier to manipulate, and involves breaking down the system to reverse‐engineer it to obtain its required constituent subsystems. It usually uses traditional workshop or microfabrication methods to develop the chiral structure through externally controlled technology, such as direct laser writing,^[^ ^30^ ^]^ lithography techniques^[^ ^31^ ^]^ and mechanically twisting.^[^ ^32^ ^]^ In particular, mechanically twisting, an age‐old yet mature technique in fiber manufacturing, has been an indispensable and effective process to construct functional materials and intelligent systems (e.g., artificial muscle,^[^ ^33^ ^]^ torsional refrigeration,^[^ ^34^ ^]^ fibrous battery electrodes^[^ ^35^ ^]^). The twisted morphology that arises during the process of fiber twisting is perfectly matches the helical fibrous morphology, which are ideal for the development of CPL materials.

Moreover, CPL materials’ emission efficiency and luminescence stability are other pivotal aspect concerning practical applications. In this regard, aggregation‐induced emission (AIE) molecules offer a significant advantage due to the enhanced luminescence efficiency in the solid state derived from their restricted intramolecular motion (RIM).^[^ ^36^, ^37^ ^]^ In addition, the improved luminescence signals of AIE molecules within the constrained environment of polymer chains can be polarized by the induction or scattering of hierarchical helical microstructures spanning the molecular and macroscale of the polymer chains, implying an optimal candidate for the fabrication of CPL.^[^ ^38^, ^39^ ^]^ As such, we envision that the marriage of polymer fiber twisting technique and AIE molecules’ attributes will facilitate the large‐scale production of solid‐state CPL materials while simultaneously ensuring the high luminescence efficiency and stability of CPL materials.

Here, we propose a continuous melt spinning combined twisting method to fabricate full‐color and tunable CPL macro‐fibers starting from AIEgens‐doped polymer, wherein the direction of fiber twist determines the handedness of CPL. Leveraging the highly controllable and adjustable twisting process, we revealed a twisting‐induced top‐down chirality transfer mechanism in which the embedded achiral AIEgens successively align with the orientation and torsional arrangement of the polymer chains under the macro twisting forces, ultimately producing chirality at the molecular level. The top‐down chirality transfer enabled the intense chiral optical activity with the *g*_lum_ of 10^−2^, which is comparable to or even surpasses that of most chiral small molecule or polymer self‐assembly systems. Moreover, the mechanical force‐induced chirality transfer method is versatile, scalable, and easy to implement, making it applicable to numerous commercial polymers and fluorophores. This enables an extensive functional CPL fiber library including full‐color and handedness‐tunable CPL fibers. Finally, thanks to the weavability of the fibers, several cryptographic patterns were embroidered on sewing machines using our CPL macro‐fibers, demonstrating their potential for a wide range of applications in the field of information encryption and anti‐counterfeiting.

## 2 Results and Discussion

### 2.1 Design and Fabrication of Twisting‐Induced CPL Fiber

**Figure 1:**
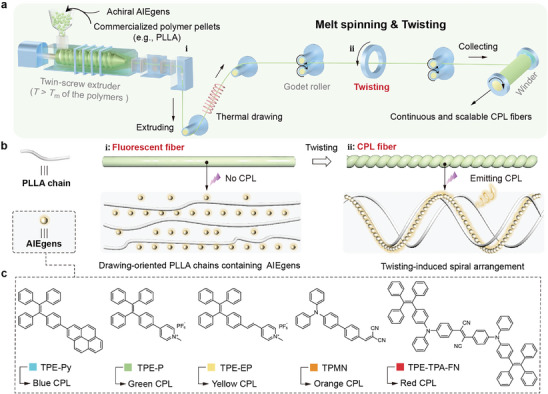
Design and fabrication of the CPL macro‐fiber. a) Schematic diagram of the melt spinning and twisting process for the manufacture of CPL macro‐fibers. b) Schematic illustration of the chirality transfer from the macro‐ to the molecular scale, in which the helical microstructures within the twisted fiber were formed, bringing out the twisting‐induced directional torsion of polymer chains and thus resulting in the chiral rearrangement of AIEgens. c) Various achiral AIEgens with full‐colored emission.

The fabrication process of the CPL macro‐fibers involves two main steps: melt spinning and twisting, as shown in **Figure** **1**a. Initially, thermoplastic polymer pellets were heated in a twin‐screw extruder to their melting temperature (*T*_m_), and then AIEgens were added to generate a homogeneous mixture. This mixture is extruded from the spinneret, forming a melt stream that is immediately cooled and cured in air to obtain the primary fibers. At this stage, the *T*_m_ of the selected polymer should be lower than the decomposition temperature (*T*_d_) of AIEgens to ensure their stable luminescent property. For this proof‐of‐concept, commercial poly (*L*‐lactic acid) (PLLA) was chosen, which not only has thermal properties compatible with AIEgens (Figure S1, Supporting Information), but is also sustainable with reduced dependence on fossil fuels and biodegradability. Following the thermal drawing process, the diameter of the primary fibers is reduced to 115 ± 15 µm (Figure S2, Supporting Information), and the PLLA chains obtain a high orientation along the fiber axis. Such highly oriented polymer chains, on the one hand, enable efficient fluorescence emission from AIEgens in the fiber matrix based on the RIM emission mechanism,^[^ ^40^, ^41^ ^]^ thus empowering the resulted fibers with excellent optical properties (Figure 1b; Figure S3, Supporting Information). On the other hand, the highly oriented architectures endow the drawn fibers with greater mechanical strength (Figure S4, Supporting Information), allowing for the subsequent twisting process to customize the helical microstructure. During mechanically twisting, the distorted PLLA chains originated from the helical torsion of fibers forced the embedded AIEgens chiral rearrangement, thereby evoking CPL activity (Figure 1b). Such chiral optical activity does not originate from the intrinsic molecular properties of achiral AIEgens, but from the “top‐down” chirality transfer from macroscopic chiral torsion to the AIEgens aggregates on the molecular level. This speculation will be thoroughly discussed in the following sections. A series of achiral AIEgens such as TPE‐Py, TPE‐P, TPE‐EP, TPMN, and TPE‐TPA‐FN, could be incorporated into the twisted PLLA fibers with full‐color CPL emission ranging from 450 to 600 nm (Figure 1c), examining the universality and efficiency of our method.

### 2.2 CPL Optical Properties of Macroscopically Twisted Fibers

**Figure 2:**
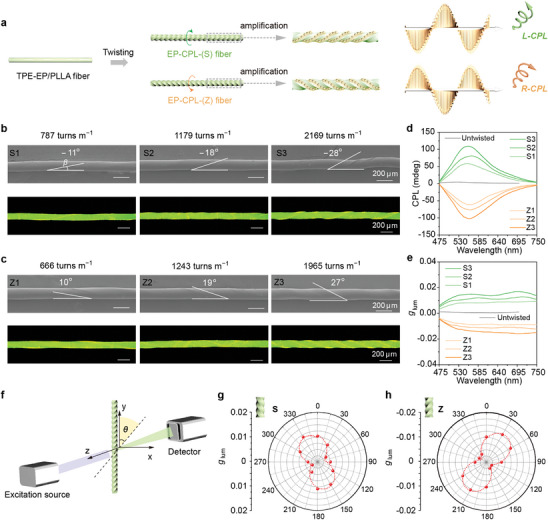
Surface morphologies and CPL optical properties of twisted Y‐fiber. a) Schematic illustration of a Y‐fiber twisted along the either S‐ or Z‐direction (Y‐fiber‐(S) or Y‐fiber‐(Z)), emitting *L*‐CPL and *R*‐CPL, respectively. b,c) SEM and fluorescence microscopy images of Y‐fiber‐(S) and Y‐fiber‐(Z) with different twist densities. d) CPL spectra and e) *g*_lum_ spectra of Y‐fiber‐(S) and Y‐fiber‐(Z) with different twist densities. f) Schematic illustration of angle‐dependent CPL measurement, where the fiber is rotated in a plane perpendicular to the incident light. g,h) *g*_lum_ angle dependence of Y‐fiber‐(S) and Y‐fiber‐(Z).

The CPL optical properties of TPE‐EP/PLLA fibers were first investigated to demonstrate the feasibility of the twisting‐induced CPL strategy. The emission spectrum of the TPE‐EP/PLLA fiber displays yellow emission (referred to as Y‐fiber) with a maximum value of 556 nm and a quantum yield of 67.8%, which is significantly higher in comparison to 57.6% for the TPE‐EP powders (Figure S3, Supporting Information). The enhanced emission is mainly due to the increased rigidity and restricted intramolecular motions of AIEgens within frozen polymer chains. For the twisted Y‐fibers, twist direction and density are crucial parameters. The twist direction is determined by the direction of the applied torque during twisting. Specifically, clockwise twisting along the fiber axis results in a spiral microstructure resembling the deflection of the letter “S,” and the twisted fibers are named Y‐fiber‐(S). On the contrary, counterclockwise twisting produces Y‐fiber‐(Z) (**Figure** **2**a). Twist density, expressed in turns per meter (turns m^−1^), was used to evaluate the degree of fiber twisting. One turn is a full rotation of the fiber around its axis, equivalent to an angular displacement of 360°. By controlling the twisting time, fibers with different twist densities were produced. All these fibers possess excellent mechanical properties (Figure S5, Supporting Information) and are therefore very robust for practical applications. In addition, the elongation at break of the fibers increases with increasing twist density, indicating an improvement in the toughness of the fibers.

The micromorphology of Y‐fiber with different twist directions and densities was analyzed using scanning electron microscopy (SEM) and fluorescence microscopy (Figure 2b,c). The fiber surface displayed clear S‐ and Z‐shaped helical microstructures. The angle between the sloping grain and the fiber axis is defined as the *β*, which represents the degree of twisting. A large angle value corresponds to a large degree of twist. As shown in Figure 2b,c, *β* increased with the twist density. For Y‐fiber‐(S), the angle value rose from −11° to −28°, and for Y‐fiber‐(Z) fibers, it increased from 10° to 27°. The twisting operation did not change the fluorescence lifetime of the fibers (Figure S6, Supporting Information). The CPL spectra of these twisted Y‐fibers are shown in Figure 2d and Figure S7 (Supporting Information). Y‐fiber‐(S) showed positive left‐handed (*L*‐) CPL signals at 556 nm, while Y‐fiber‐(Z) emitted negative right‐handed (*R*‐) CPL signals, showing the mirror‐image CPL spectra. Moreover, the CPL signal intensity improved as the twist density increased. To quantify the magnitude of CPL, the *g*_lum_ was calculated using the equation, *g*_lum_ = 2 × (*I*_L_ − *I*_R_)/(*I*_L_ + *I*_R_), where *I*_L_ and *I*_R_ denote the intensities of *L*‐CPL and *R‐*CPL, respectively. The value of *g*_lum_ exhibits the same tendency along with the elevated twist density like the CPL signal intensity, reaching a maximum value of 1.5 × 10^−2^ (Figure 2e). The perfect mirror symmetry of the CPL signals and *g*_lum_ spectra of Y‐fiber‐(Z) and Y‐fiber‐(S) with similar twist density but opposite chirality demonstrates the reproducibility and controllability of the twisting‐induced CPL strategy. However, for untwisted Y‐fibers, although the PLLA polymer chains can transfer their molecular chirality to the luminophore of TPE‐EP based on the intermolecular interactions between two constitutes (Figures S8 and S9, Supporting Information),^[^ ^42^ ^]^ they almost showed silent CPL signal, and the *g*_lum_ value (10^−4^) is two orders of magnitude lower than that of our twisted fiber (Table S1, Supporting Information) and can be ignored. Therefore, we hypothesize that the twisted fibers with helical microstructure dominate the chiroptical activity of achiral TPE‐EP molecules rather than PLLA polymer chain chirality. Moreover, a variety of CPL fibers with precisely customized handedness and *g*_lum_ can be prepared by varying the twist density and direction (Figures S10 and S11, Supporting Information), offering more opportunities to dig out the relationships between the helical structure and CPL property. The results indicate the possibility of enantioselectively CPL controlling by modulating the macroscopic mechanical twisting direction of the fibers.

Furthermore, considering the angle dependence of CPL in anisotropic samples,^[^ ^43^, ^44^ ^]^ a series of angle‐dependent CPL measurements of the twisted Y‐fibers were carried out. Before gathering the data, the tested twisted Y‐fiber was fixed on a custom‐built sample stage and rotated in the plane perpendicular to the incident light (Figure 2f; Figure S12, Supporting Information). As a result, the highly anisotropic profile of the altitudinal polar coordinate of *g*_lum_ versus the fiber rotation angle *θ* was observed in Figure 2g,h, exhibiting varying anisotropy depending on the fiber twisting direction. As shown in Figure 2g and Figure S13 (Supporting Information), the maximum value of *g*_lum_ of Y‐fiber‐(S) is ≈0.01 at a *θ* of 150° and 330°, while the minimum value is ≈0.002 at *θ* = 60° and 240°. For Y‐fiber‐(Z), the maximum and minimum values of *g*_lum_ are ≈ −0.01 at *θ* = 30° and 210° and −0.002 at the *θ* of 120° and 300°, respectively (Figure 2h; Figure S14, Supporting Information). These angle‐dependent *g*_lum_ values are primarily attributed to the linear birefringent properties of the fibers (Figures S15 and S16, Supporting Information).^[^ ^45^ ^]^ When the electromagnetic waves emitted from AIEgens pass through the birefringent material, they are decomposed into two orthogonally propagating waves with different speeds of propagation due to different intrinsic refractive index in the two orthogonal directions within the anisotropic medium. A phase delay was generated between these two waves after leaving the birefringent medium, which would affect the polarized wave detected by the instrument.^[^ ^46^, ^47^ ^]^ Moreover, the linear polarization characteristic indicates the high‐oriented feature of the AIE luminophores along the fiber axis induced by PLLA chains.

### 2.3 Insights Into Twisting‐Induced Chirality Transfer Mechanisms

**Figure 3:**
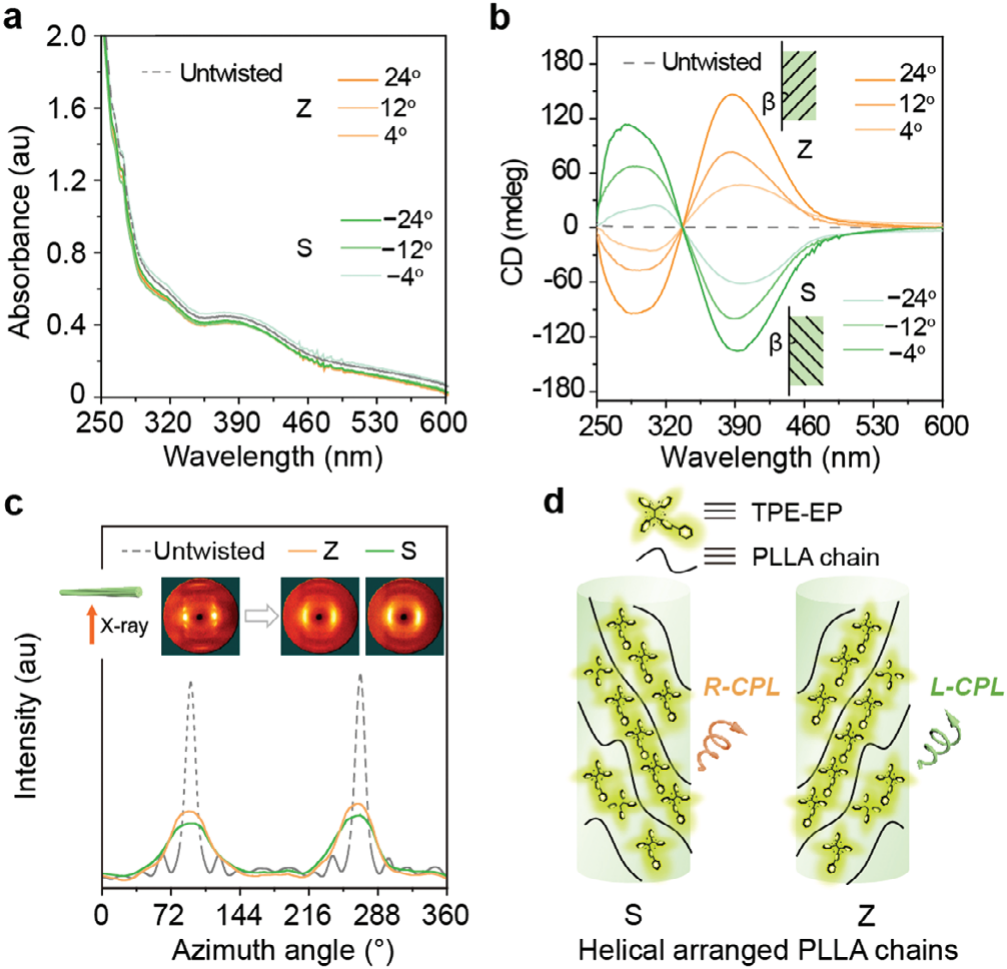
Molecular chirality in twisted Y‐fibers. a) UV/Vis spectra and b) corresponding CD spectra of untwisted fiber models and Z‐twisted or S‐twisted fiber models with different twists. c) Azimuthal profiles of the 2D‐WAXS intensities obtained from Y‐fiber‐(Z), Y‐fiber‐(S), and the untwisted Y‐fibers. Insets: Corresponding 2D WAXS patterns. d) Schematic illustration of chirality transfer at the molecular level, showing that twisting forces in different directions lead to helical rearrangements of the polymer chain and AIE dye in the corresponding chiral directions.

To investigate the mechanism behind these twisting‐induced CPL properties, we first performed circular dichroism (CD) spectroscopy tests to probe the chirality of the AIE molecules in the ground state within twisted fibers. Due to strong light scattering, the tested twisted Y‐fiber with a diameter of approximately hundred microns exhibited linear polarization artifacts during CD testing. Therefore, we prepared a twisted fiber model with a larger diameter of 7 mm to cover the incident spot of the CD spectrometer to avoid light scattering. **Figure** **3**a,b show clear absorbance and bisignated CD response of TPE‐EP in Y‐fiber‐(Z) and Y‐fiber‐(S) models, whereas TPE‐EP in untwisted fibers exhibits no CD signal. It is observed from the CD spectrum of Y‐fiber‐(Z) that a negative split‐type Cotton effect is induced, with a negative Cotton effect at 304 nm, followed by positive Cotton effect at 396 nm, attributed to the characteristic absorption band of TPE‐EP molecule between 250 and 600 nm. Moreover, the CD intensity increases as |*β*| rises from 4° to 24°. Meanwhile, Y‐fiber‐(S) with reverse handedness shows a mirror CD profile and a similar trend of increasing CD intensity with |*β*|. These CD results indicate that the achiral AIE molecules indeed capture the chirality from the twisted fiber host. We infer that macroscopic mechanical twisting force cause directional rearrangements of PLLA chains and AIEgens within the fibers at the molecular level and induce the formation of chiral helical structures at the microscopic scale.

To further support this speculation, we investigated the orientation of the PLLA chains before and after twisting using 2D wide‐angle X‐ray scattering (2D WAXS). In Figure 3c, the 2D WAXS image of the untwisted Y‐fibers exhibits clear low‐angle diffraction arcs at the equator, indicating that the PLLA chains are aligned along the fiber axis. Upon twisting the fibers along the S or Z direction, the arc length increases, indicating a weakening of the axial alignment of the PLLA chains and the emergence of helically oriented architecture.^[^ ^48^, ^49^ ^]^ The degree of orientation was further calculated according to the equation: (360°−*H*)/360°, where *H* is the full width of the half‐maximum of the arc integral curve of the Debye ring diffraction at the equator.^[^ ^50^ ^]^ As expected, the degree of molecular chain orientation in Y‐fiber‐(Z) and Y‐fiber‐(S) is estimated to be 73%–76%, lower than that of untwisted Y‐fibers (≈94%) (Table S2, Supporting Information), verifying the formation of the helical orientation of the PLLA chains after twisting. Overall, our results demonstrate the applicability of the twisting‐induced top‐down chirality transfer mechanism to build helical microstructures in fiber through macroscopic twisting forces, and enable achiral AIEgens in the fiber host to obtain molecular‐level chiral properties (Figure 3d). The simplicity and practicality of inducing CPL and controlling handedness of AIEgen‐incorporated‐twisted fibers is fascinating compared to bottom‐up molecular manufacturing techniques.

### 2.4 Versatility of Twisting‐Induced Top‐Down Chirality Transfer

**Figure 4:**
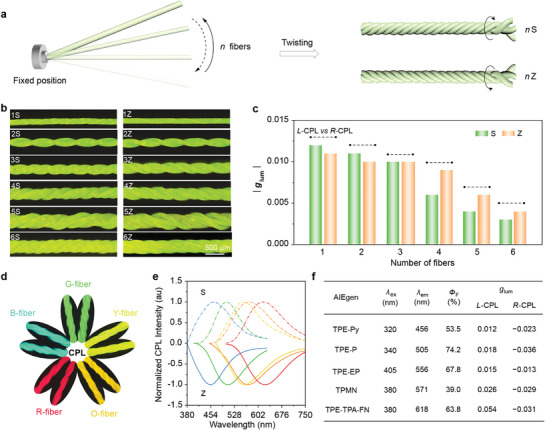
Versatility of twisting‐induced CPL strategy. a) Schematic illustration of a twisted fiber bundle composed of multiple Y‐fibers, where *n* represents the number of fiber strands and S or Z represents the twist direction. b) Fluorescence microscopy images of Y‐fiber‐(S) bundles or Y‐fiber‐(Z) bundles composed of 1–6 strands of fibers. c) *g*_lum_ spectra of twisted fiber bundles in S‐ or Z‐direction. d) Fluorescence microscopy images of multicolor CPL fiber bundles with S‐ or Z‐twist direction obtained from co‐twisting of 3 fibers. e,f) Mirrored CPL spectra and optical properties of the multi‐color CPL fiber bundles described above.

It is known that almost all commercial fiber products are prepared through co‐twisting processes to improve their weavability in textile production. Thus, we investigated the availability of our twisting‐induced top‐down chirality transfer mechanism in more complex situations. As illustrated in **Figure** **4**a, multiple Y‐fibers were arranged in parallel and then co‐twisted along the S or Z direction to produce a twisted fiber bundle. The morphology of the single fibers in the co‐twisted fiber bundle shows obvious helical deformation due to the mutual extrusion between the twisted fibers (Figure 4b). Therefore, the resulting fiber bundles possessed a clear mirrored CPL signal similar to a single twisted Y‐fiber, but the |*g*_lum_| value decreased with increasing fiber number while the mirrored symmetry of the CPL signal is also weakened (Figure 4c; Figures S17–S19 and Table S3, Supporting Information). The decrease in |*g*_lum_| value is due to the complicated process of mechanical stresses transfer within the fiber bundles, which reduces the twisting degree of the helical microstructures. Consequently, the helical alignment of PLLA molecular chains and TPE‐EP molecules is compromised. The reduction in mirror symmetry of the CPL signal may be attributed to the differences in properties (e.g., diameters) between individual fibers within the fiber bundle (Figure 4b), thus inducing the non‐uniform forces within the fibers during the twisting process. This may further lead to uneven twist insertion within individual fibers, thereby reducing the stability of the CPL performance of the final twisted fiber bundle. These results demonstrate the top‐down chirality transfer depends on the interaction between operating parameters (torsion direction, twist density, and number of twisted fibers), ensuring CPL generation with maximum *g*_lum_ of 10^−2^, which is comparable to or even higher than the previously reported composite systems.^[^ ^45^, ^51^, ^52^, ^53^ ^]^

Four other AIEgens with emission covering the entire visible spectrum were selected as achiral luminescent units, namely TPE‐Py, TPE‐P, TPMN, and TPE‐TPA‐FN, and were respectively incorporated into twisted fiber bundles to produce full‐color CPL emissions. In addition to the yellow light emitted by the above‐mentioned TPE‐EP, these AIEgens emitting blue, green, orange, and red luminescence, respectively (Figure S20, Supporting Information). The resulting CPL fiber bundles were named B‐, G‐, Y‐, O‐, and R‐fibers according to their emission colors. All these fiber bundles show dazzling fluorescence of different colors under the radiation of UV light and maintain high luminescence efficiency due to the AIE effect (Figure 4d). Moreover, AIEgens‐doped twisted fibers with opposite twist directions exhibit mirror‐image signal from 456 to 618 nm, covering full‐color CPL emission (Figure 4e). Besides, the *g*_lum_ values of the full‐color CPL fiber bundles consistently remained the same order of magnitude of 10^−2^ (Figure 4f; Figure S21, Supporting Information).

Moreover, we substituted the fiber matrix with poly(vinyl alcohol) (PVA) and poly (propylene carbonate) (PPC). These AIEgens‐dopped twisted fiber bundles also exhibited CPL activity (Figure S22, Supporting Information). In addition, we also found that even ordinary commercial fluorescent fibers can be endowed with CPL functions as long as they are macroscopically twisted (Figure S23, Supporting Information). These results further strongly demonstrate the versatility of our twisting strategy for producing CPL fibers or fiber bundles.

### 2.5 Embroidery Encryption Application

**Figure 5:**
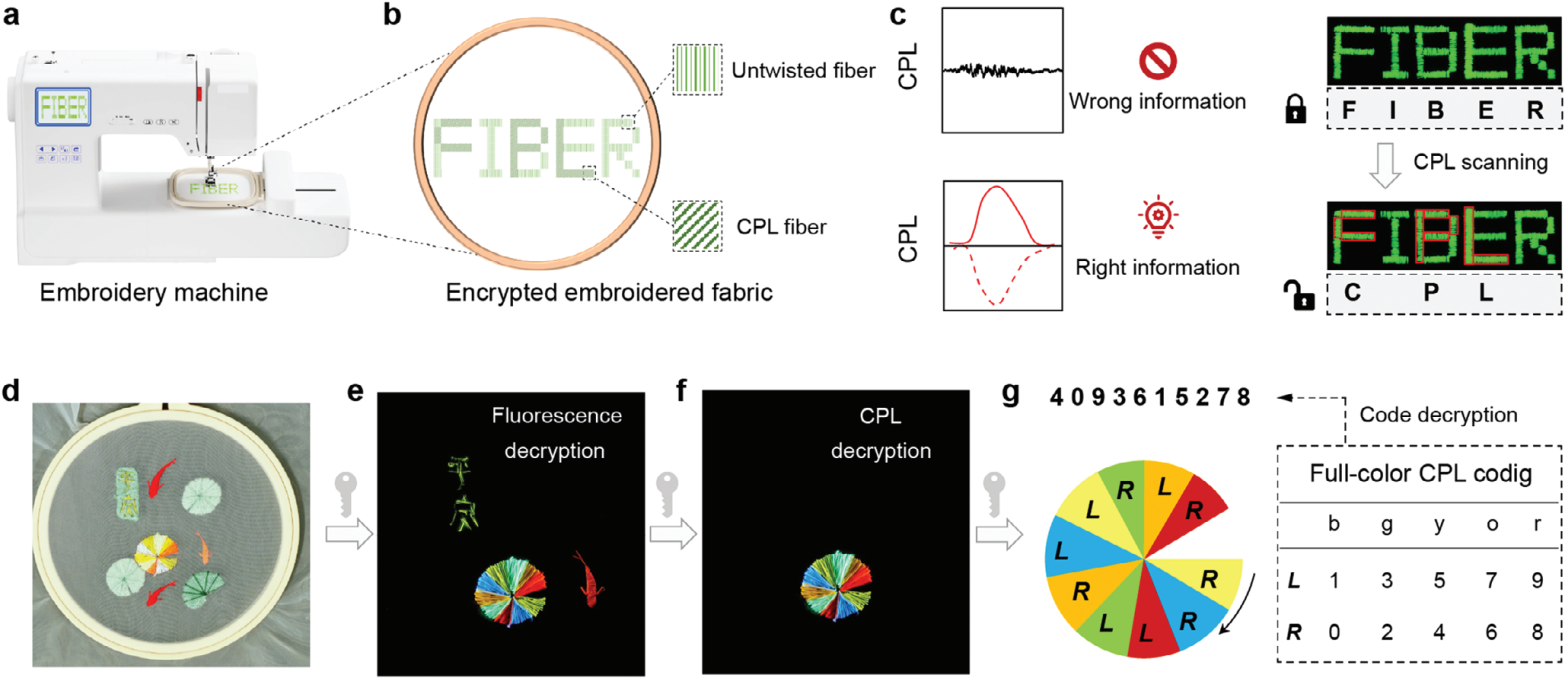
Encryption applications of CPL fiber bundles. a) A cartoon representation of the encrypted information created during the embroidery process. b) An image of encrypted “FIBER” using CPL fiber bundles and untwisted fibers. c) Information encryption using CPL scanning. d–g) Information encryption using fluorescence, CPL, and emission color codes of CPL fiber bundles.

Given the fact that we have successfully integrated fluorescent and circularly polarized signals into co‐twisted fiber bundles, a variety of multi‐level encryption modes based on CPL attributes were described by embroidery techniques. **Figure** **5**a,b illustrated a fabric embroidered with a word using the twisted and untwisted fluorescent fiber bundles. These fiber bundles appear identical under normal light and UV exposure. Directly reading the information results in a false message of “FIBER.” However, CPL scanning revealed information encoded with CPL activity while filtering out non‐CPL fluorescence signals, emitting the true message “CPL” (Figure 5c). In addition, as previously stated, we have developed full‐color CPL fiber bundles leveraging the versatility of the twisting‐induced CPL strategy. Thus, integrating full‐color emission characteristics into fluorescence and CPL signals would offer more significant advantages in information encryption. For example, Figure 5d,e displayed lotus pond fish embroidery, in which non‐fluorescent fiber bundles are first concealed under UV irradiation. Consequently, the Chinese character “平安”, the small fish, and the colorful lotus leaf plate are clearly visible. However, when scanning the canvas with a CPL scanner, it was found that only the lotus leaf plate was embroidered with CPL fibers (Figure 5f). Nevertheless, this image still lacks sufficient information and necessitates the use of a codebook that has been previously compiled for interpretation. As illustrated in Figure 5g, the emission wavelength and chiral direction were used to predefine and assign a code for each color block of the lotus leaf. By reading the corresponding color and CPL clockwise from the notch of the lotus leaf, the encrypted digital information represented by the ten‐digit code “4093615278” can be decrypted. Such fiber encryption pattern that incorporates an embroidery design is much more complex than traditional encryption patterns because it utilizes fluorescent, CPL, and emissive color‐related embroidery pattern coding, providing a more secure encryption scheme.

## 3 Conclusion

In summary, we presented a twisting‐induced top‐down chirality transfer mechanism for the development of macroscopic CPL fiber, which offers a well‐documented solution to the long‐standing challenge of scalable fabrication and application of CPL materials. Various polymers like PLLA, PVA, and PPC combined with achiral AIEgens were readily processed into CPL fibers by carefully controlling the twisting parameters in space and time. Based on the CD, 2D WAXS, and CPL results, we uncovered that the chiral optical properties are derived from chiral transfer from macroscopic mechanical twisting to AIE molecular aggregates on the molecular level. This top‐down chirality transfer allows for the possibility of controlling enantioselectively molecular arrangements via modulation of the operating parameters (torsion direction, twist density, number of twisted fibers, etc.) of the twisting process, thereby offering a versatile, controllable, and scalable means of fabricating CPL materials in extensive achiral luminescent systems. In addition, full‐color CPL fibers could be achieved by changing luminophores, whose weavability facilitates designing multilevel encryption patterns using embroidery techniques. This work opens new pathways for the development of chiral optical materials, significantly contributing to their application in various fields such as information encryption, 3D display, optoelectronic devices, asymmetric synthesis, and biomedical applications.

## 4 Experimental Section

A detailed experimental section can be found in the Supporting Information.

## Conflict of Interest

The authors declare no conflict of interest.

## Author Contributions

X.Y. and Y.C. designed the research project and X.Y. carried out the experiments and analyzed the data under the supervision of Y.C., X.Z., and M.Z. Q.L., X.L., and Z.Q. helped to analyze the data. L.C. drew the mechanism diagram. X.Y. wrote the manuscript; X.Z. and Y.C. revised the manuscript. All authors discussed, revised, and approved the manuscript to be published.

## Supporting information



Supporting Information

## Data Availability

The data that support the findings of this study are available in the supplementary material of this article.
