# Supplementary material for: Mechanically Twisting‐Induced Top‐Down Chirality Transfer for Tunable Full‐Color Circularly Polarized Luminescent Fibers
Source: Adv Sci (Weinh). 2024 Dec 4;12(4):2412778. doi: 10.1002/advs.202412778 (PMC11775519; doi:10.1002/advs.202412778)
Supplement: Supplementary file 1 — Supporting Information [file ADVS-12-2412778-s001.docx]

Supporting Information

Mechanically Twisting-Induced Top-Down Chirality Transfer for Tunable Full-color Circularly Polarized Luminescent Fibers

Xiaoxiao Yu, Linfeng Chen, Qin Liu, Xiaoqing Liu, Zhenduo Qiu, Xinhai Zhang*, Meifang Zhu and Yanhua Cheng*

State Key Laboratory for Modification of Chemical Fibers and Polymer Materials, College of Materials Science and Engineering, Donghua University, Shanghai, 201620 (P. R. China)

**Table of Contents**

| 1. | *Materials and Methods* | S3 |
| --- | --- | --- |
| 2. | *Figures and Tables* | S5 |
|  | *2.1 Basic thermodynamic properties of TPE-EP and PLLA* | S5 |
|  | *2.2 AIE properties of TPE-EP* | S6 |
|  | *2.3 Mechanical and photophysical properties of Y-fiber* | S7 |
|  | *2.4 Chirality of PLLA polymer* | S9 |
|  | *2.5 CPL handedness and g_lum_ tailoring* | S10 |
|  | *2.6 Angle dependence of CPL signal* | S11 |
|  | *2.7 Birefringence of twisted Y-fibers* | S12 |
|  | *2.8 Helical arrangement of PLLA chains in twisted Y-fibers* | S13 |
|  | *2.9 CPL activity of twisted fiber bundles* | S14 |
|  | *2.10 Photophysical properties and g_lum_ of full-color CPL fibers* | S16 |
|  | *2.11 CPL activity of twisted fibers with different polymer matrices* | S17 |
|  | *2.12 CPL activity of commercially twisted fluorescent fibers* | S18 |
| 3. | *References* | S19 |

1. ***Materials and Methods***

***1.1 Materials***

PLLA was purchased from Nantong Jiuding Biological Engineering Co. LTD. Full-color AIEgens—TPE-Py, TPE-P, TPE-EP, TPMN, TPE-TPA-FN and TPE-OH, were purchased from AIEgen Biotech Co., LTD. PVA and PPC were purchased from Aladdin, Co., Shanghai, China and Shanghai Macklin Biochemical Technology Co., Ltd, respectively. All starting materials were used as received. Before preparing the fibers, the polymer was uniformly mixed with AIEgen in an organic solvent. The polymer was doped with 0.5 wt% concentration of AIEgen for all samples, according to our previous work^[1]^. Taking TPE-EP/PLLA as an example, PLLA was dissolved in chloroform to create a homogeneous solution with a concentration of 20 mg mL^−1^. Then, the TPE-EP dissolved in THF was added to the PLLA solution and stirred for 30 minutes. The resulting mixture was evaporated at room temperature and dried in a vacuum oven to obtain the final TPE-EP/PLLA composite material.

***1.2 Preparation of twisted-induced CPL fiber***

Melt-spinning technology was used to fabricate AIEgens-doped PLLA fibers and AIEgens-doped PPC fibers. Specifically, the twin-screw extruder temperature was set at 180 °C for PLLA fiber extrusion and 160 °C for PPC fiber extrusion. The collection rate was 10.0 m min^−1^. The virgin fibers were drawn under tension through heated rollers at 65°C to obtain twistable strength. Wet-spinning technology was used to fabricate AIEgens-doped PVA fibers. The resulting AIEgen/polmber fibers were dried in an oven at 60 °C for 12 hours before subsequent twisting.

Twisting is achieved by securing the fiber at one end and using a twister at the other end to apply a clockwise or counterclockwise twisting force along the fiber axis. The rotating speed of the twisting machine is 300 r min^−1^, and different fiber twisting densities are obtained by controlling the twisting time. During the twisting process, tension is applied to both ends of the fiber to prevent the fiber from unraveling or rolling on itself. Once the twisting insertion process is complete, the fiber ends are securely bolted to prevent any torque release. To make a twisted fiber bundle, multiple fibers are arranged side-by-side, and a twisted fiber bundle is obtained by fixing one end of the bundle and applying a twist to the other end.

***1.3 Characterization***

Scanning electron microscopy (SEM) images were obtained on Hitachi SU8010 SEM. Optical photographs were recorded with a Canon EOS 80D camera. Fluorescent images were taken by a fluorescent microscope (Nikon Eclipse Ni-U). Photoluminescence (PL) spectra, circular dichroism (CD) spectra and fluorescence quantum yields (QY) were measured on Horiba FluoroMax Plus spectrofluorometer, Applied Photophysics Chirascan V100, Quantaurus-QY C11347-11, respectively. CPL measurements were conducted on a JASCO CPL-300. Before testing, both ends of the fiber sample were fixed on a home-made fiber holder. Thermal analyses by differential scanning calorimetry (DSC) and thermogravimetry (TG) were conducted using TA DSC Q20 and Mettler TGA2 in N_2_ atmosphere at a heating rate of 10 ℃ min^−1^, respectively. The mechanical properties of the fibers were characterized on a universal testing machine (Instron 5969). Two-dimensional wide-angle X-ray scattering (2D WAXS) experiments were performed in transmission mode on a Bruker D8 Discover system equipped with a Cu Kα target. Polarized micrographs were taken with a Nikon Eclipse Ni-U microscope equipped with crossed polarizers. The grayscale value of the image is calculated by first obtaining the red (*R*), green (*G*), and blue (*B*) values through the Matlab program,^[2]^ and then calculating according to the following formula: Grayscale = 0.299**R* + 0.587**G* + 0.114**B*.^[3]^

1. ***Figures and Tables***

***2.1 Basic thermodynamic properties of TPE-EP and PLLA***


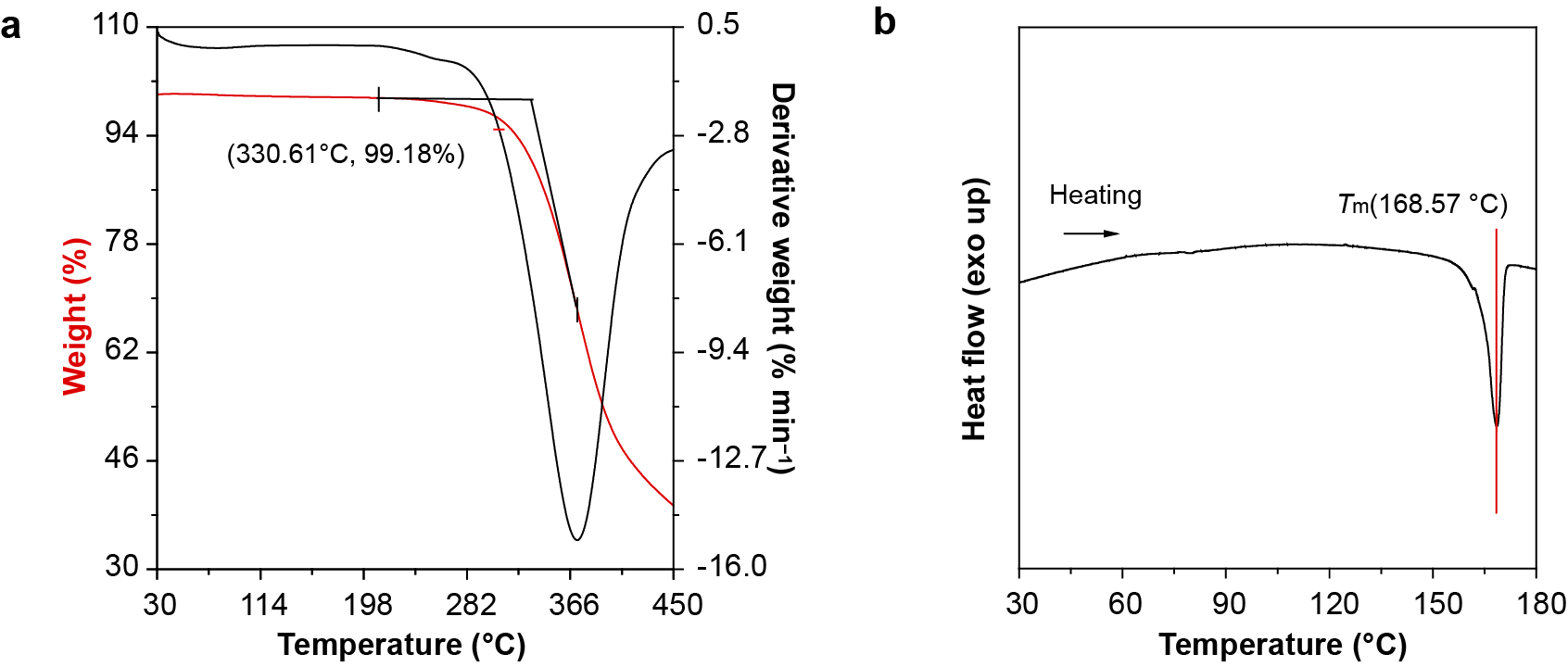


**Figure S1. Characterization of thermodynamic properties**. a) The thermogravimetric (TG) curve and first-order derivative of TPE-EP, showing that it starts to decompose at *T*_d_ = 330 °C. b) Differential scanning calorimetry (DSC) curve of first heating of PLLA, indicating melting point *T*_m_ = 168 °C.


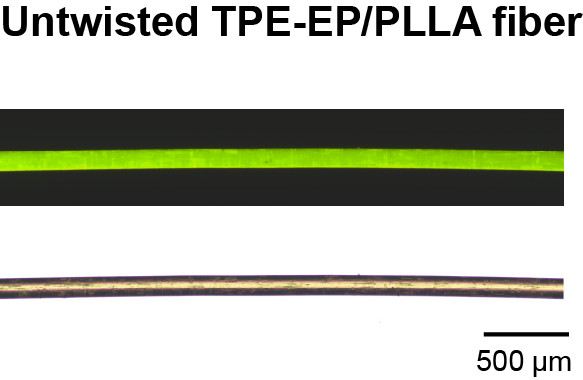


**Figure S2. Fluorescence (top) and optical micrographs (bottom) of untwisted TPE-EP/PLLA fibers.** The surface morphology of untwisted TPE/PLLA fiber appears smooth and flat.

- 1. ***AIE properties of TPE-EP/PLLA fiber***


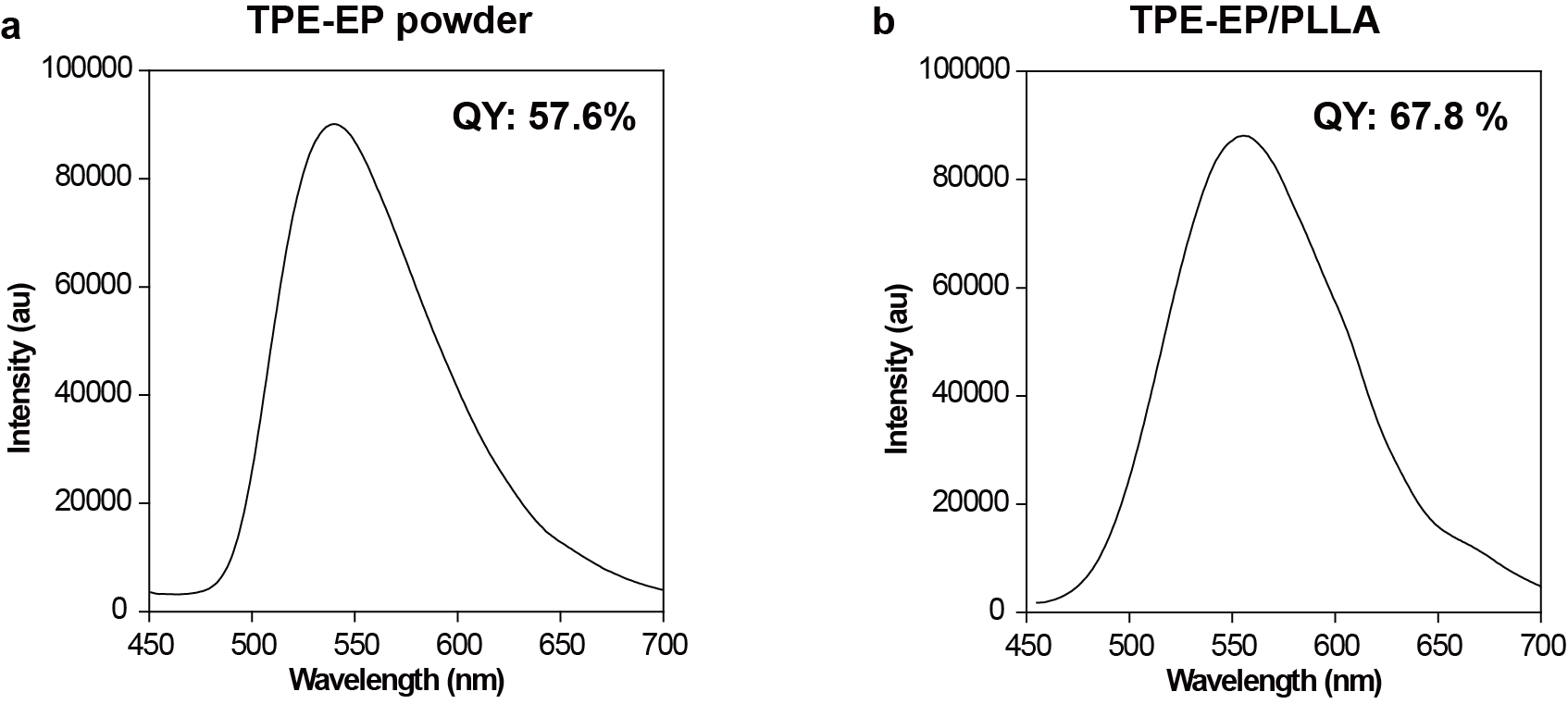


**Figure S3. Fluorescence properties of TPE-EP before and after blending with PLLA**. PL spectrum of (a) solid TPE-EP powder and (b) TPE-EP/PLLA fiber, both showing high emission intensity. The quantum yield (QY) of TPE-EP/PLLA fiber is about 10% higher than that of TPE-EP powder.


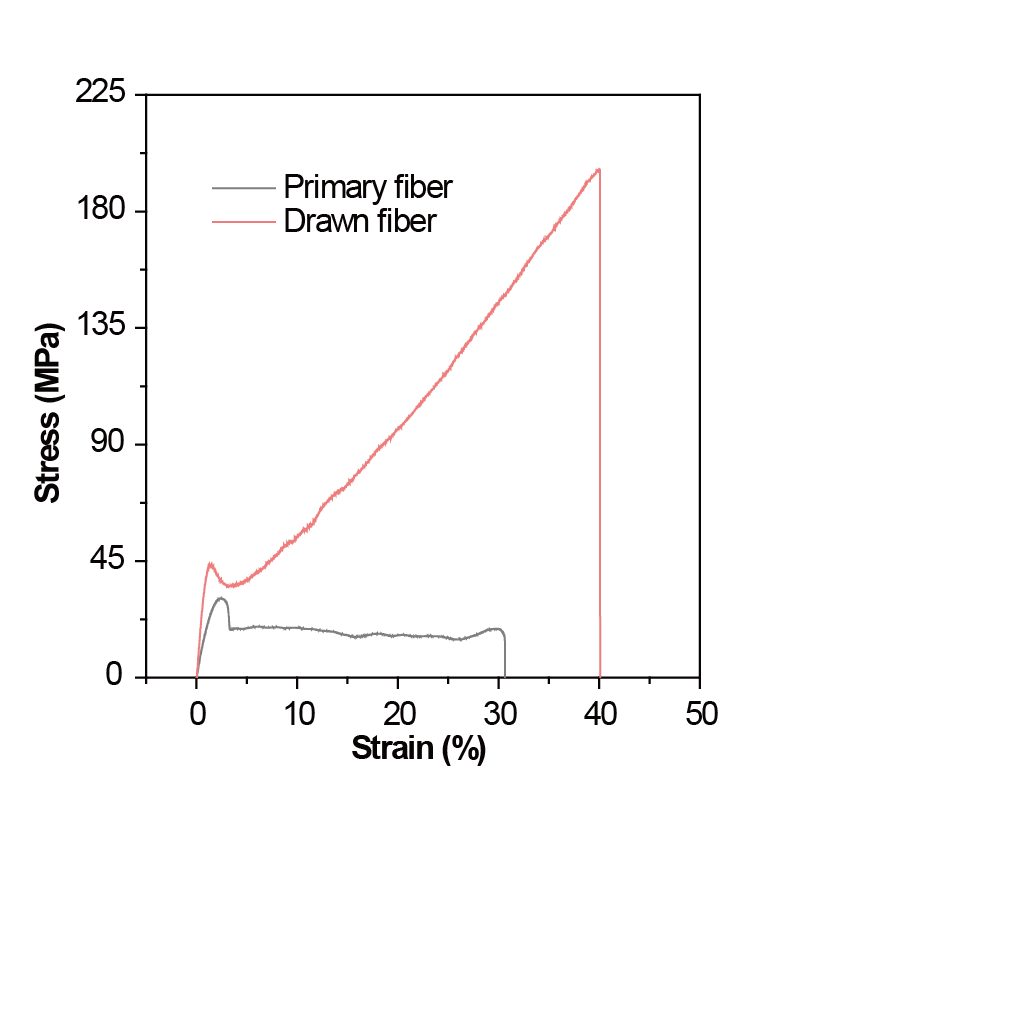


**Figure S4. Mechanical properties of TPE-EP/PLLA fibers**. Stress-strain curves of TPE-EP/PLLA fiber before and after drawing.

- 1. ***Mechanical and photophysical properties of Y-fiber***

***
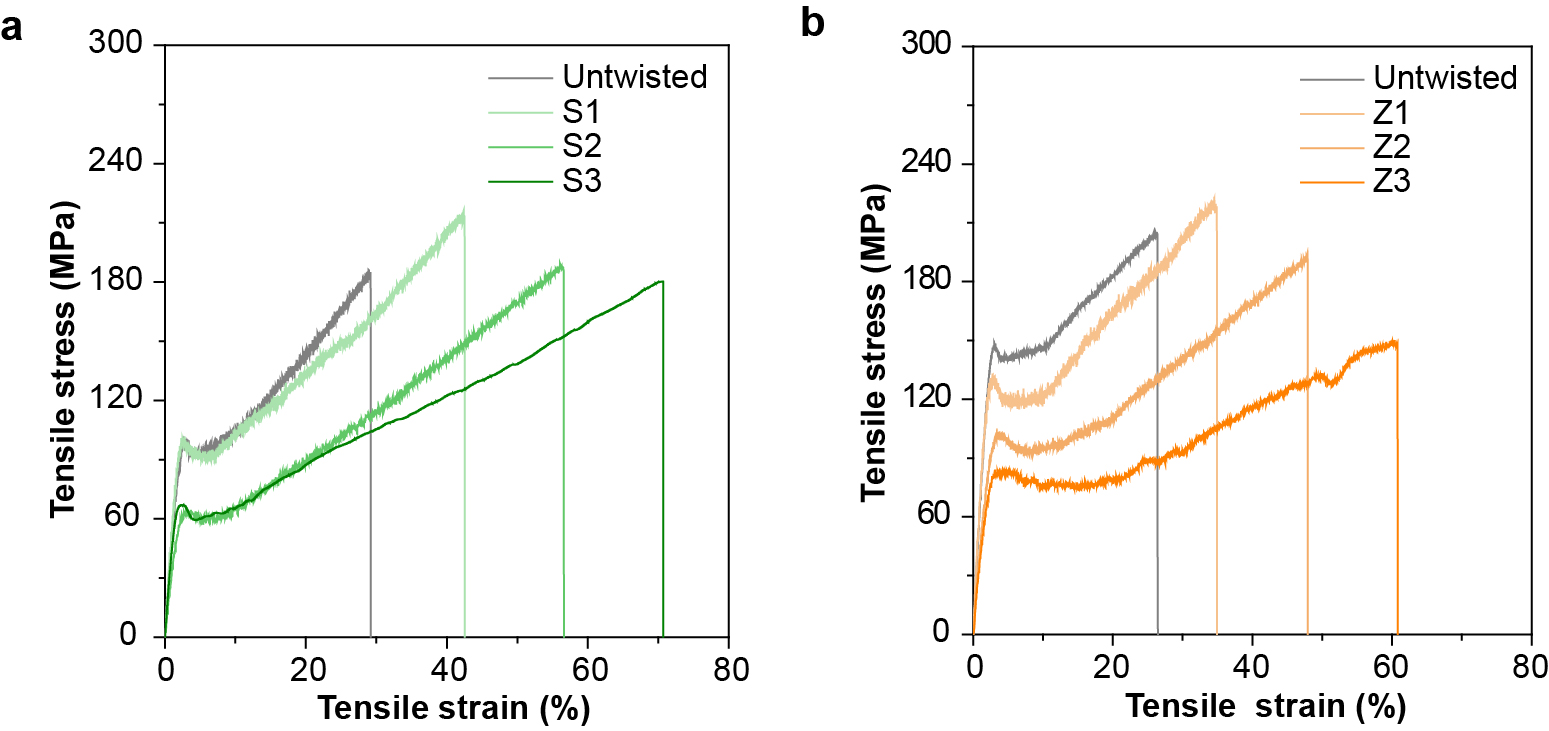
***

**Figure S5. Tensile stress-strain curves of (a) Y-fiber-S and (a) Y-fiber-Z.** The elongation at break of the fibers increases with increasing twist, while the elastic modulus decreases. The twisting treatment results in increased flexibility of the fibers, thereby improving the mechanical properties of the original fiber.

***
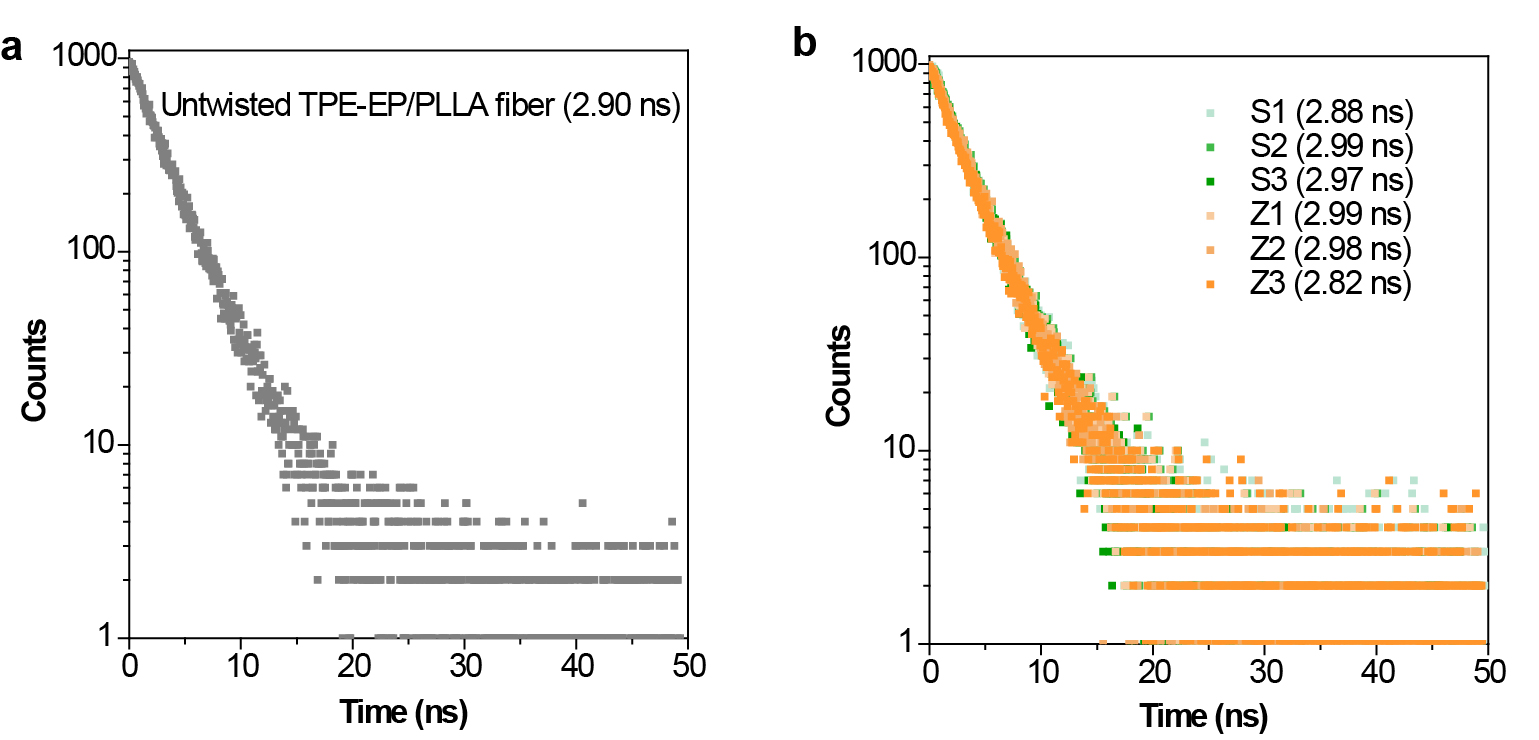
***

**Figure S6. Fluorescence decay profile of (a) untwisted TPE-EP/PLLA fiber and (b) twisted Y-fibers with different twist densities and twist directions.**

**
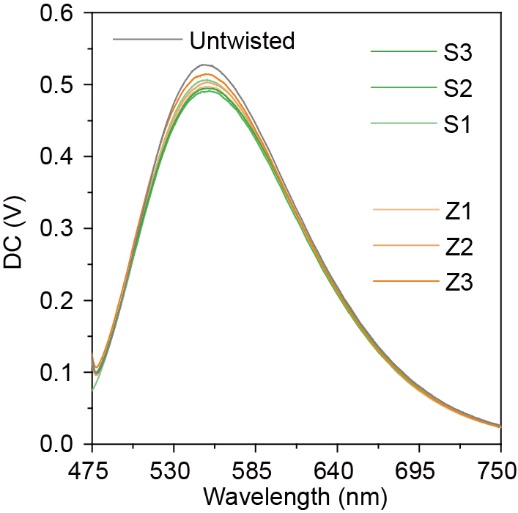
**

**Figure S7. DC spectra of Y-fiber-(S) and Y-fiber-(Z) with different twist densities.**

- 1. ***Chirality of PLLA polymer***


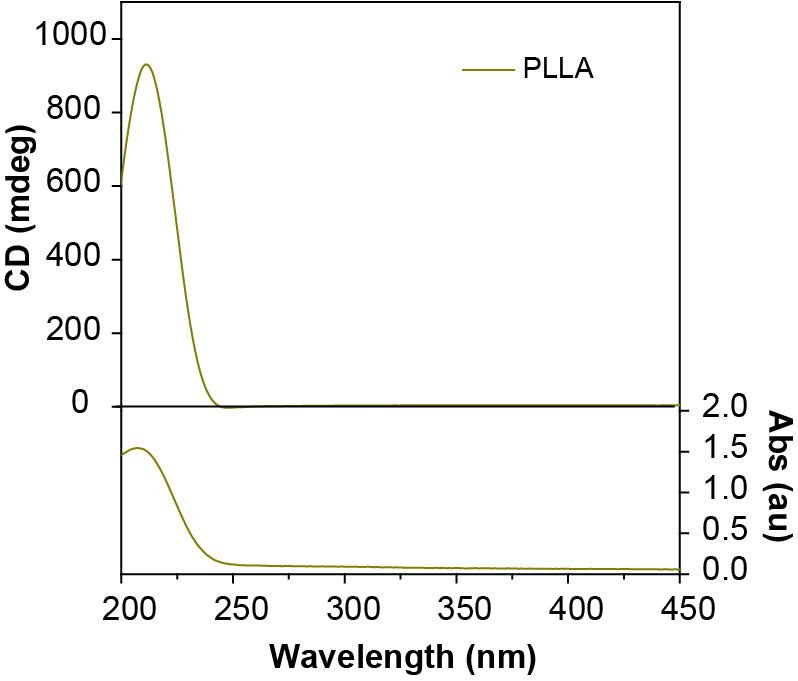


**Figure S8. CD and UV-Vis absorbance spectra of pure PLLA.** Pure PLLA exhibits the cotton effect at 210 nm due to its molecular chirality.


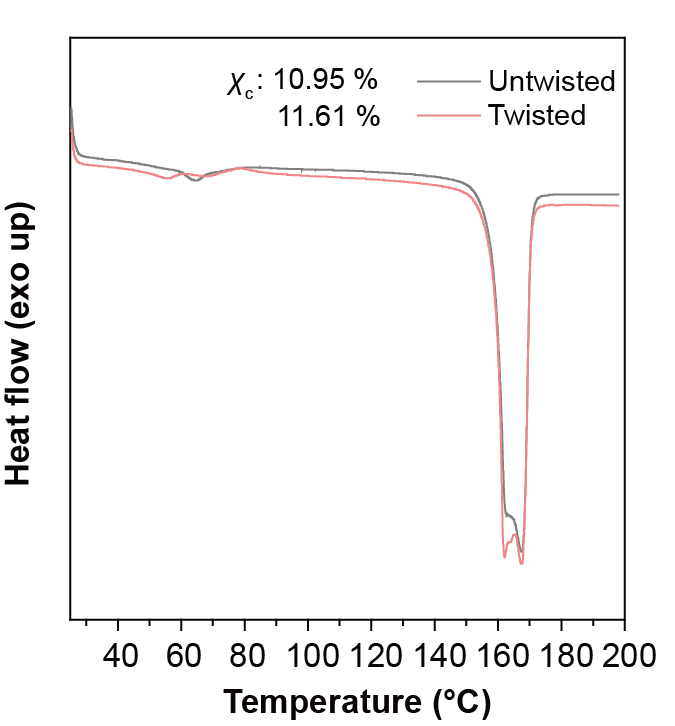


**Figure S9. DSC spectra of Y-fibers before and after twisting.** As previously reported, crystalline PLLA could act as a polarizing mediator for incorporated TPE-EP molecules, thereby generating CPL^[4]^. The crystallinity of the polymer fibers before and after twisting remained unchanged, thus ruling out the hypothesis that the strong CPL activity observed in the twisted fibers originated from PLLA polymer crystals.

***2.5 CPL handedness and g_lum_ tailoring***

**Table S1. *g*_lum_ of twisted Y-fibers with various twist directions and twist densities.**


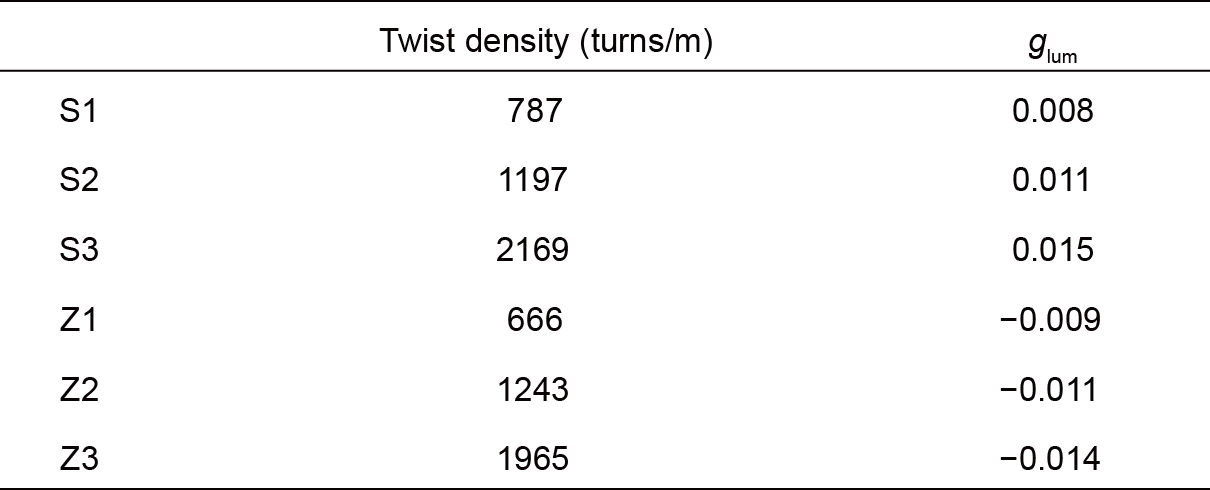


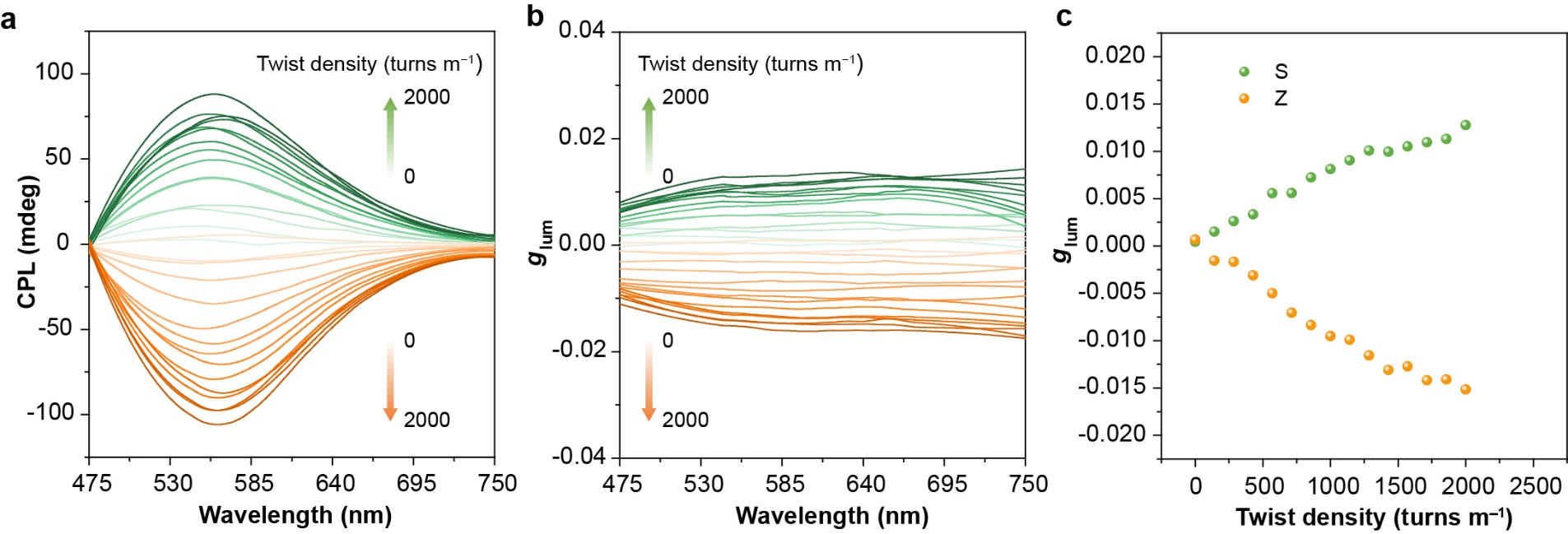


**Figure S10. CPL handedness and *g*_lum_ controlled by twist direction and twist density.** a) CPL spectra and b) *g*_lum_ spectra of Y-fiber-(S) and Y-fiber-(Z) with twist densities increasing from 0 to 2000 turns m^−1^. c) Plot of *g*_lum_ versus fiber twist density. Positive and negative CPL are achieved by changing the fiber twist direction. CPL intensity and |*g*_lum_| value increase with increasing fiber twist density.


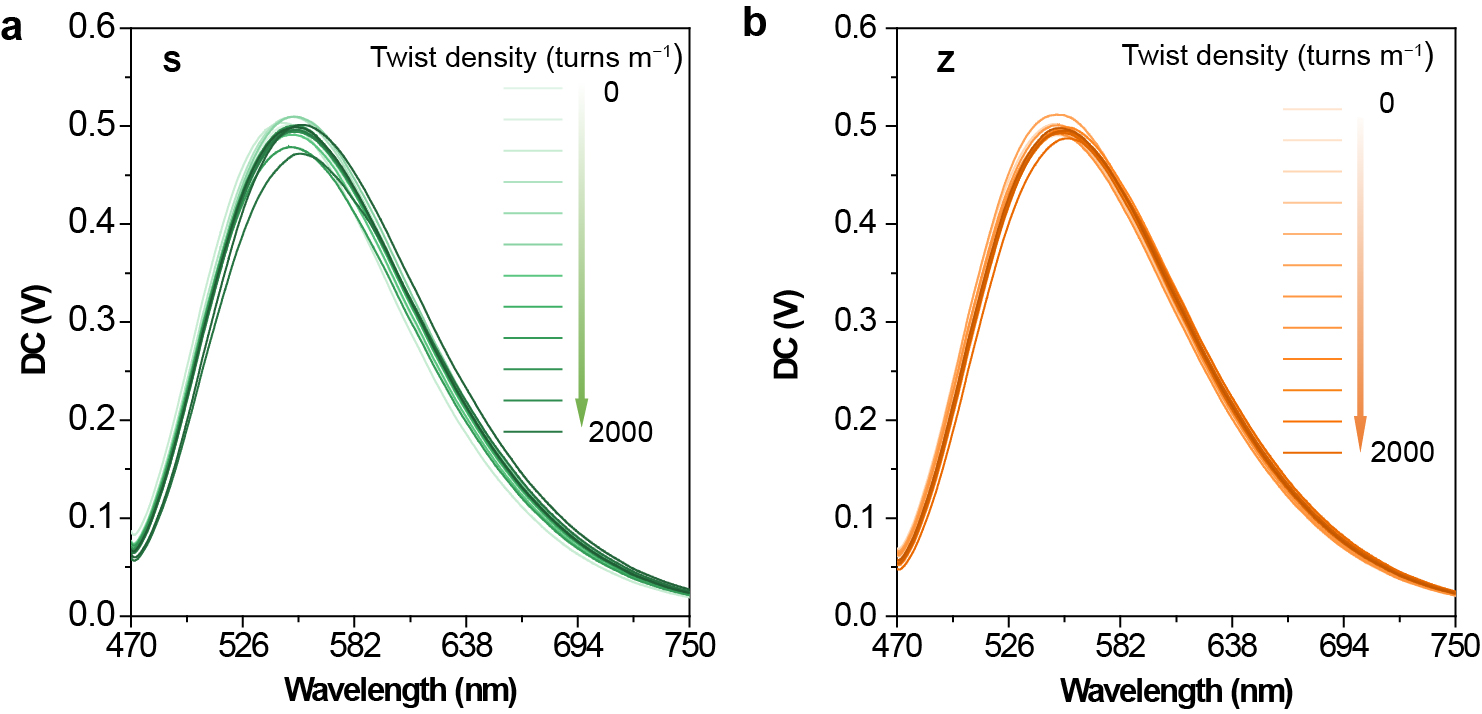


**Figure S11. DC spectra of (a) Y-fiber-(S) and (b) Y-fiber-(Z) with twist densities increasing from 0 to 2000 turns m^−1^.**

- 1. ***Angle dependence of CPL signal***


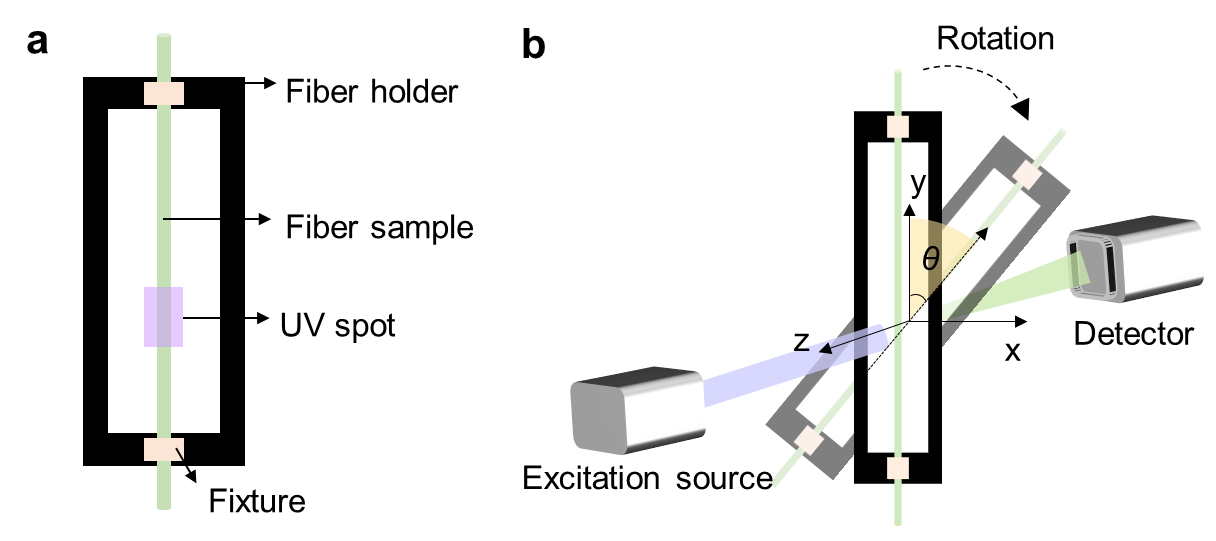


**Figure S12. Schematic diagram of CPL testing of fiber samples.** a) Schematic representation of a fiber sample mounted on a sample holder. b) Schematic diagram of the optical path and sample placement position for CPL testing. The fiber was placed perpendicular to the incident light path and the angular dependence of the CPL was measured by rotating the fiber sample in a plane perpendicular to the incident light.


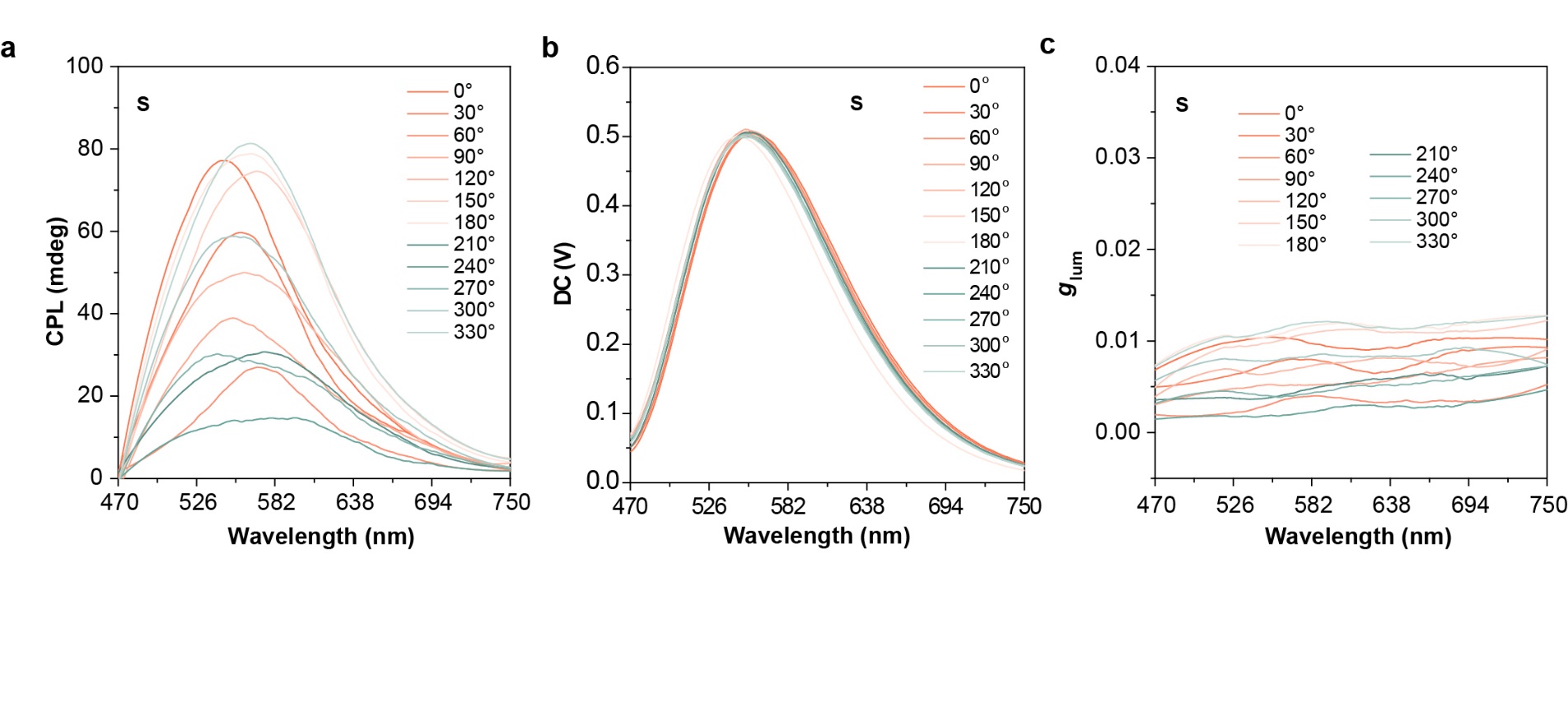


**Figure S13. Angle dependence of CPL signal of Y-fiber-(S).** a) CPL spectra, b) DC spectra and c) *g*_lum_ spectra of Y-fiber-(S) at different rotation angles from 0° to 330°.


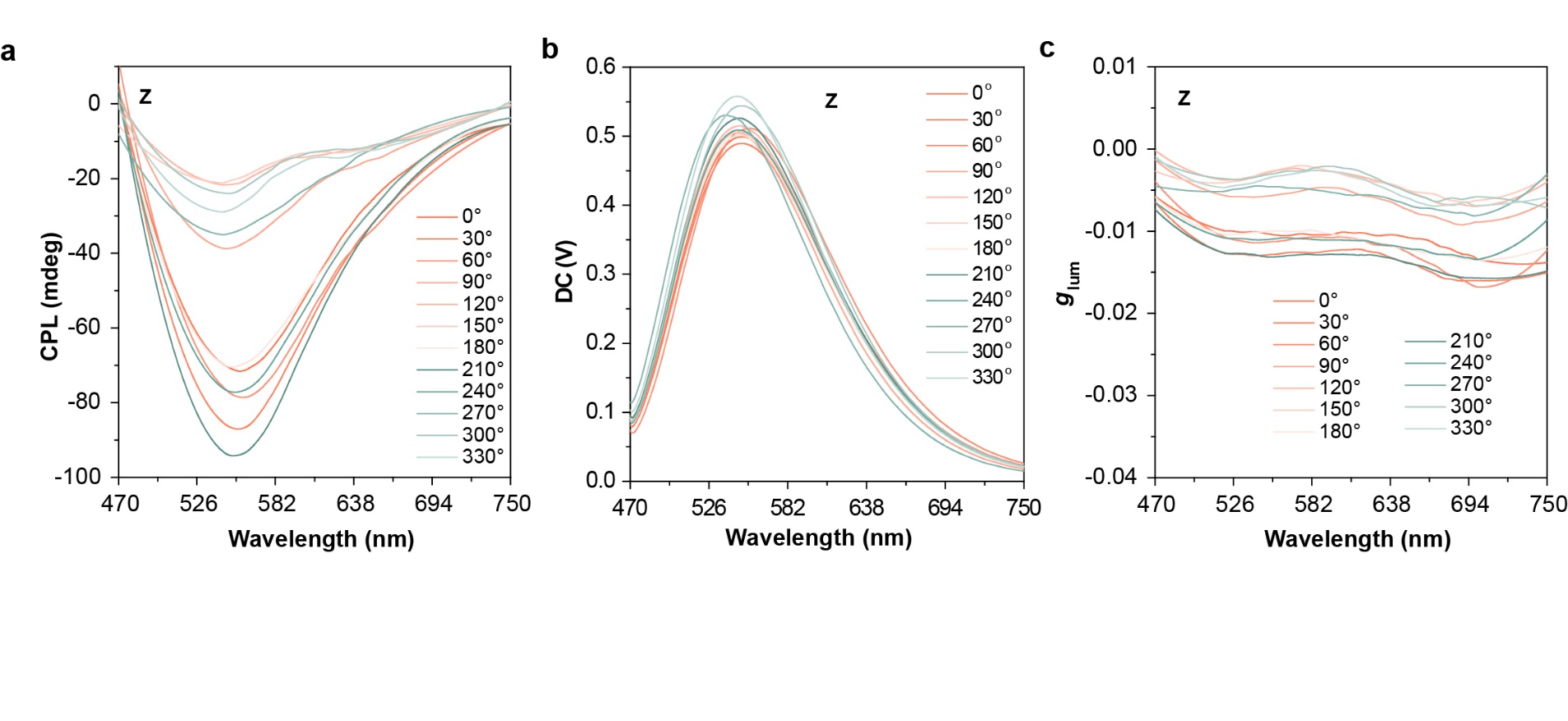


**Figure S14. Angle dependence of CPL signal of Y-fiber-(Z).** a) CPL spectra, b) DC spectra and c) *g*_lum_ spectra of Y-fiber-(Z) at different rotation angles from 0° to 330°.

***2.7 Birefringence of twisted Y-fibers***


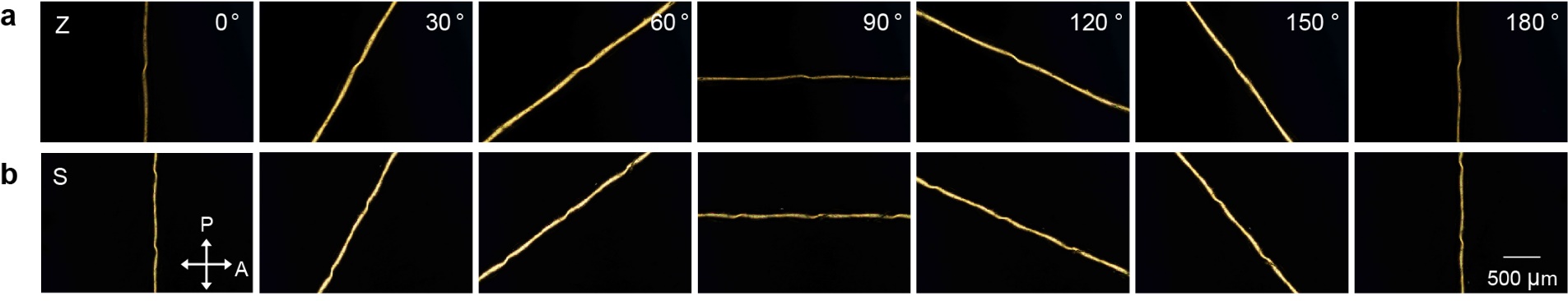


**Figure S15. Birefringence property of twisted Y-fibers.** Optical images of (a) Y-fiber-(Z) and (b) Y-fiber-(S) under polarizing microscopy using crossed polarizers. The white arrow indicated the polarization directions of the polarizer (P) and analyzer (A), respectively. The rotation angles of the objective stage are 0°, 30°, 60°, 90°, 120°, 150°, 180°, respectively. As the objective table rotates, the brightness of the images changes greatly.


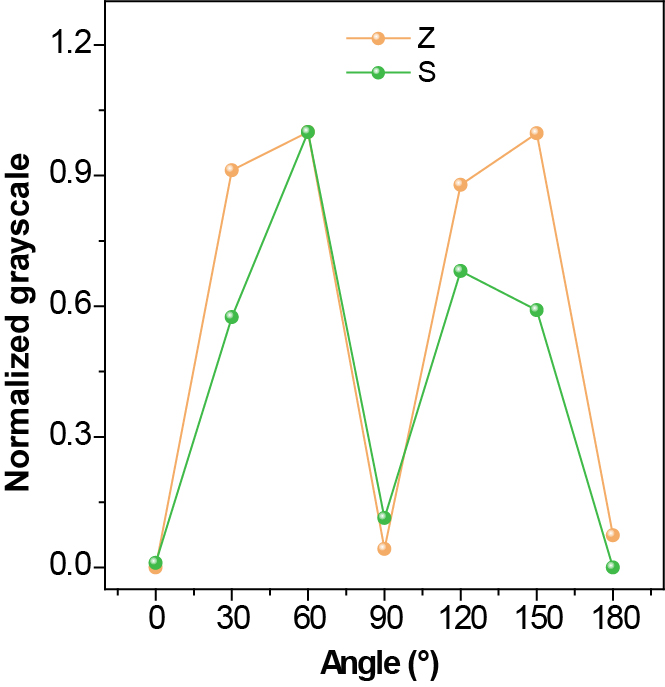


**Figure S16. Normalized grayscale of polarized optical image of twisted Y-fibers versus fiber rotation angle.** The grayscale is extracted from the images at different rotation angles in Figure S15. When the twisted Y-fiber is parallel or perpendicular to the polarizer, the image exhibits the least grayscale. Meanwhile, when the angle between the fiber and the polarizer is 30°, 60°, 120°, and 150°, the grayscale of the image becomes significantly larger. The aforementioned results demonstrate the uniaxial orientation of twisted Y-fibers.

***2.8 Helical arrangement of PLLA chains in twisted Y-fibers***

**Table S2. The degree of fiber orientation of untwisted Y-fiber, Y-fiber-(Z) and Y-fiber-(S).**


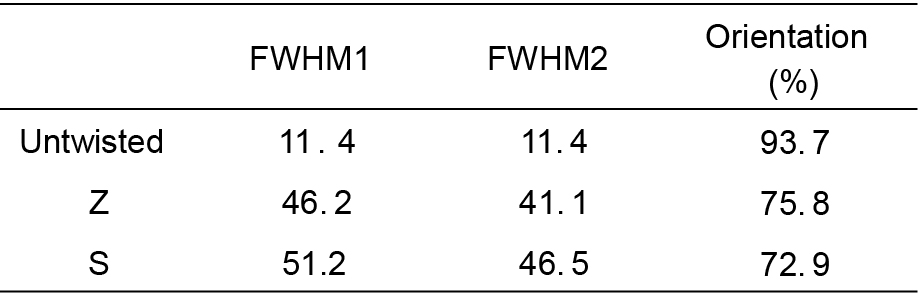


***2.9 CPL activity of twisted fiber bundles***


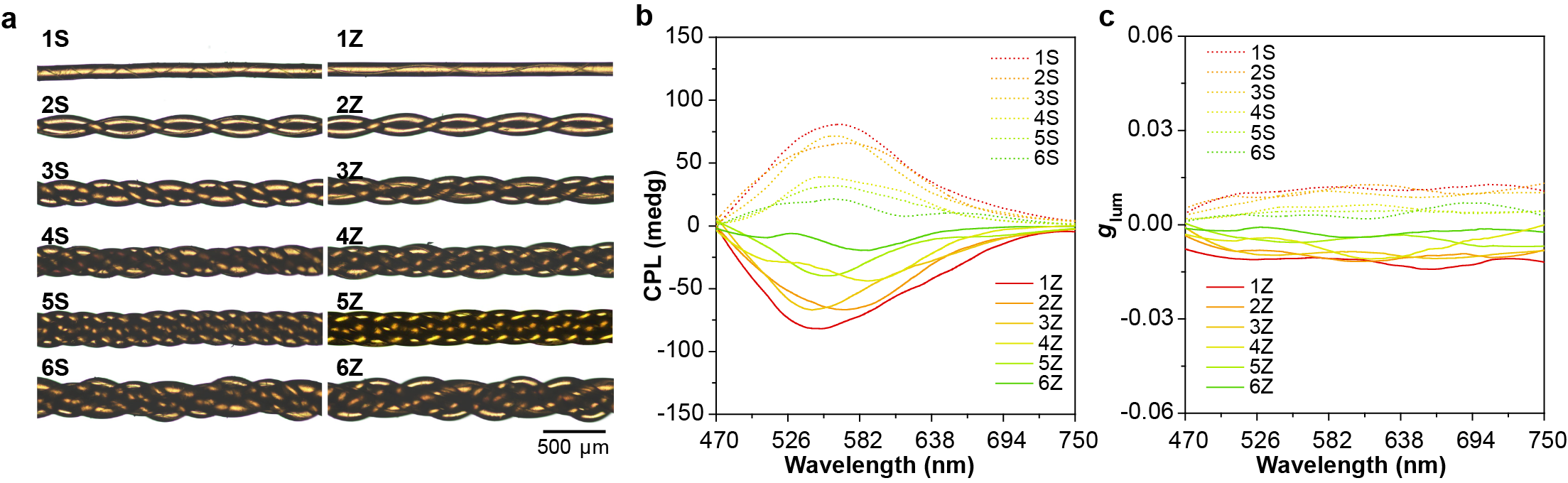


**Figure S17. Optical micrographs of S- or Z-twisted fiber bundles formed by co-twisting 1−6 fiber strands.**


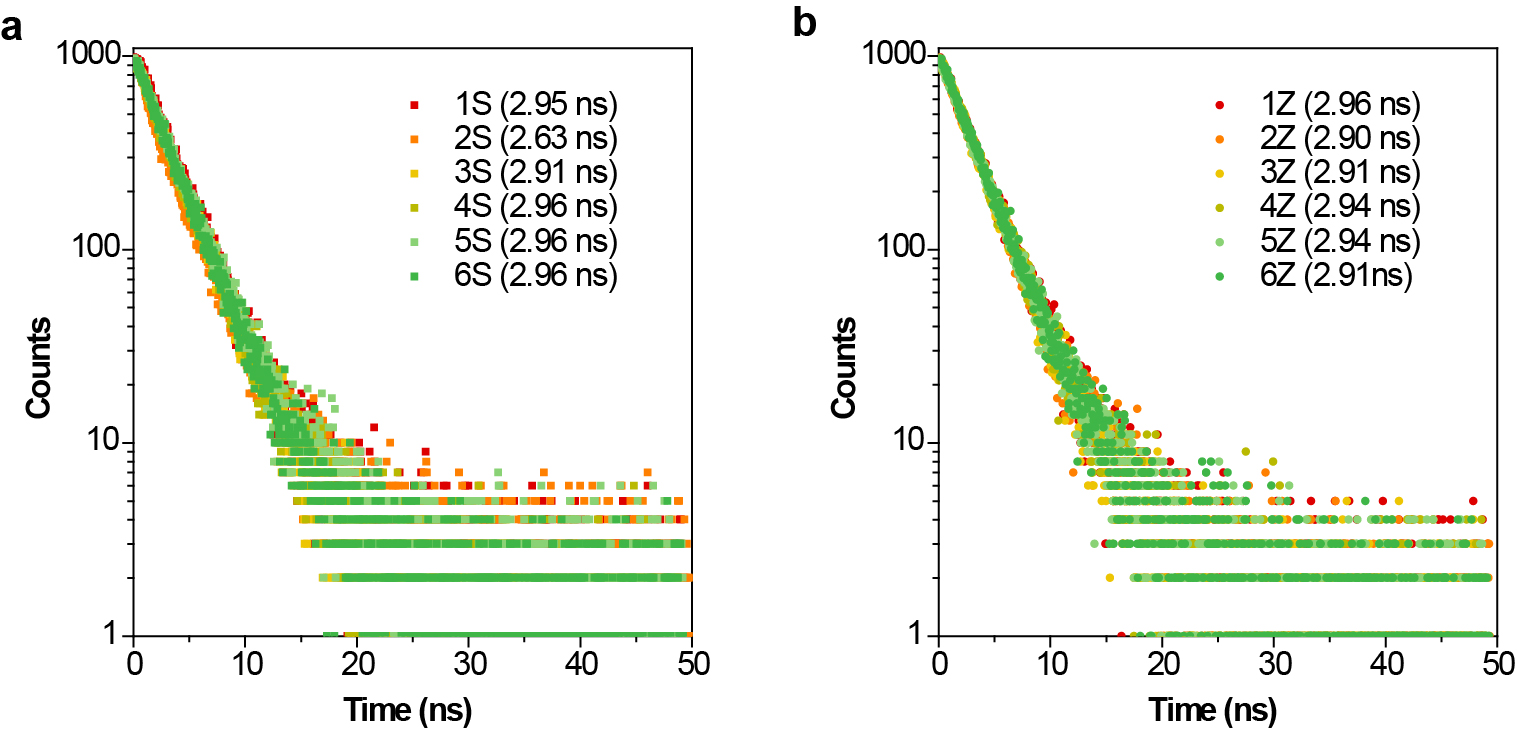


**Figure S18. Fluorescence decay profile of (a) S- or (b) Z-twisted fiber bundles formed by co-twisting 1−6 fiber strands.**


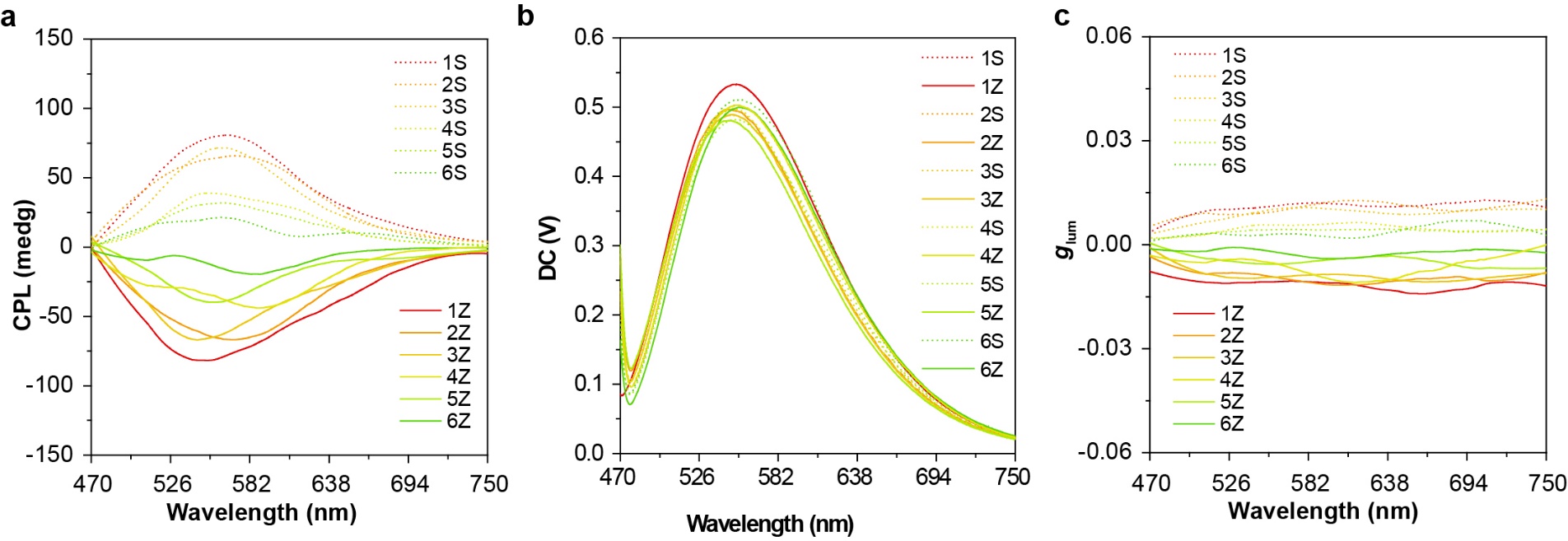


**Figure S19. CPL properties of twisted fiber bundles to demonstrate the versatility of the twisting induced CPL strategy.** a) CPL spectra, b) DC spectra and corresponding c) *g*_lum_ spectra of fiber bundles twisted in the S- or Z- direction.

**Table S3. Optical properties of twisted Y-fiber bundles**


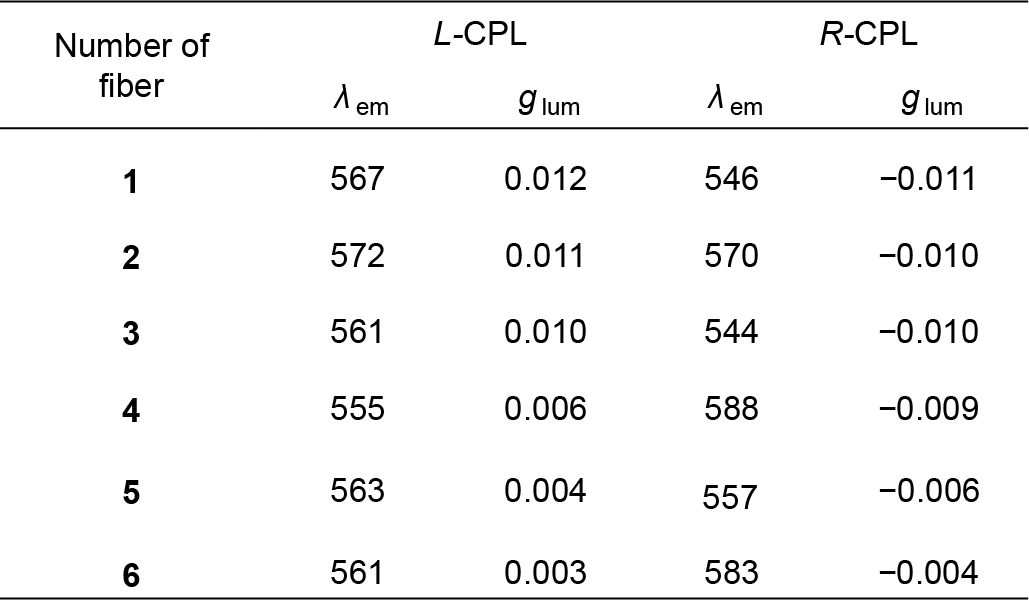


***2.10 Photophysical properties and g_lum_ of full-color CPL fibers***


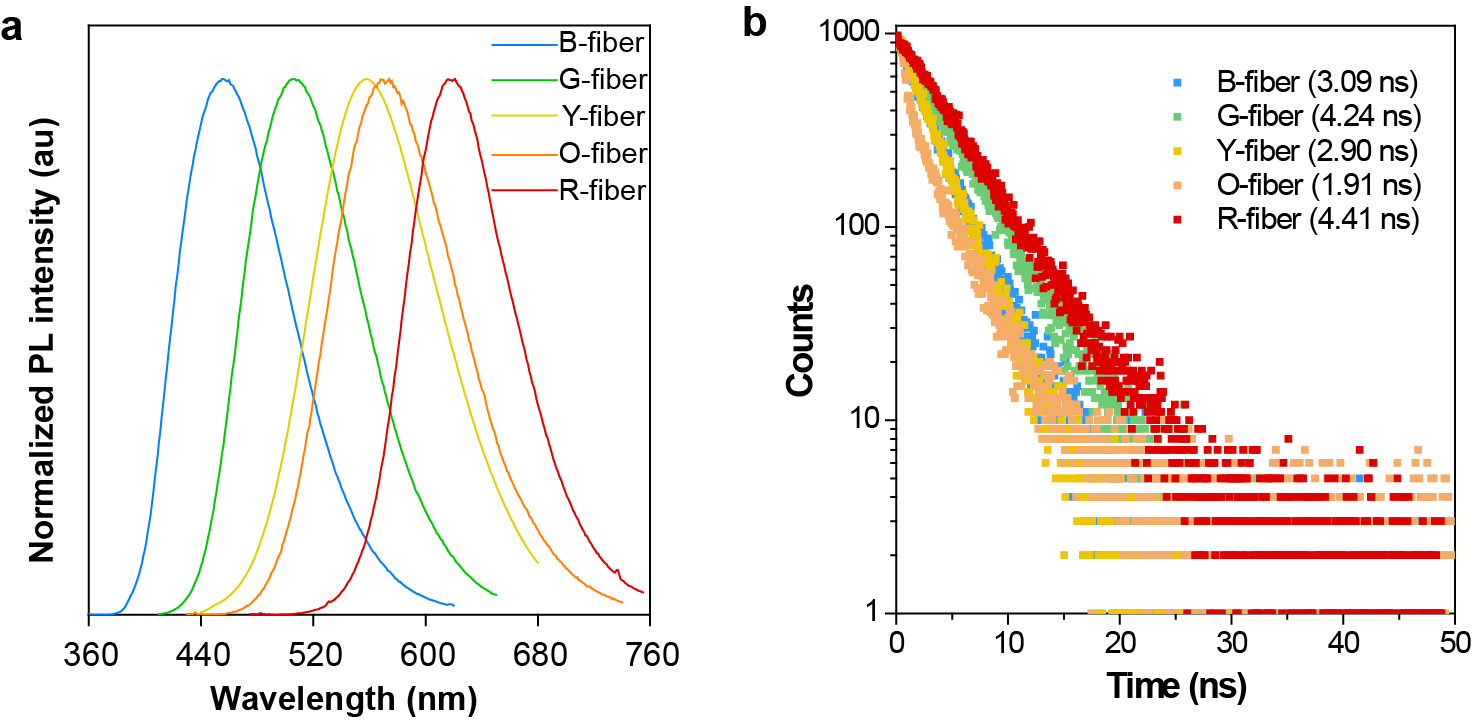


**Figure S20. a) Normalized PL spectra and b) fluorescence decay profile of full-color AIEgens-doped PLLA fibers.** It is worth noting that, B- (blue color, *λ*_em_ = 456 nm), G- (green color, *λ*_em_ = 505 nm), Y- (yellow color, *λ*_em_ = 556 nm), O- (orange color, *λ*_em_ = 571 nm) and R-fiber (red color, *λ*_em_ = 618 nm) respectively represents TPE-Py-doped, TPE-P-doped, TPE-EP-doped, TPMN-doped and TPE-TPA-FN-doped PLLA fiber.


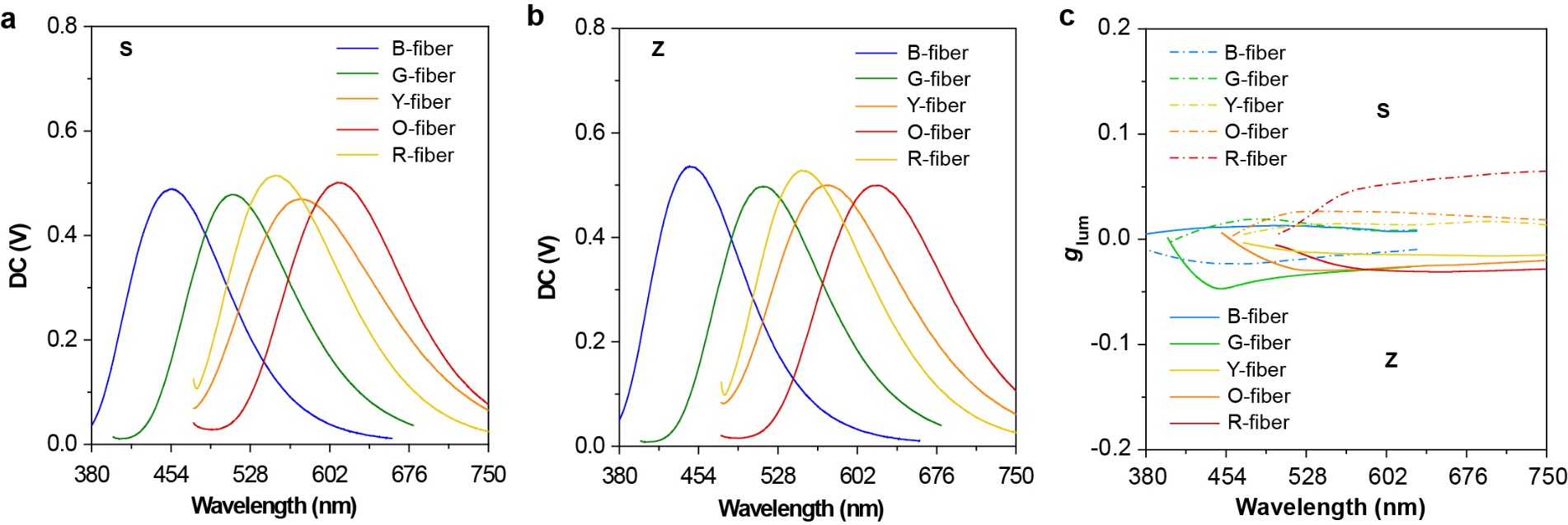


**Figure S21. a, b) DC value and c) *g*_lum_ spectra of multi-color CPL fiber bundles.**

***2.11 CPL activity of twisted fibers with different polymer matrices***

***
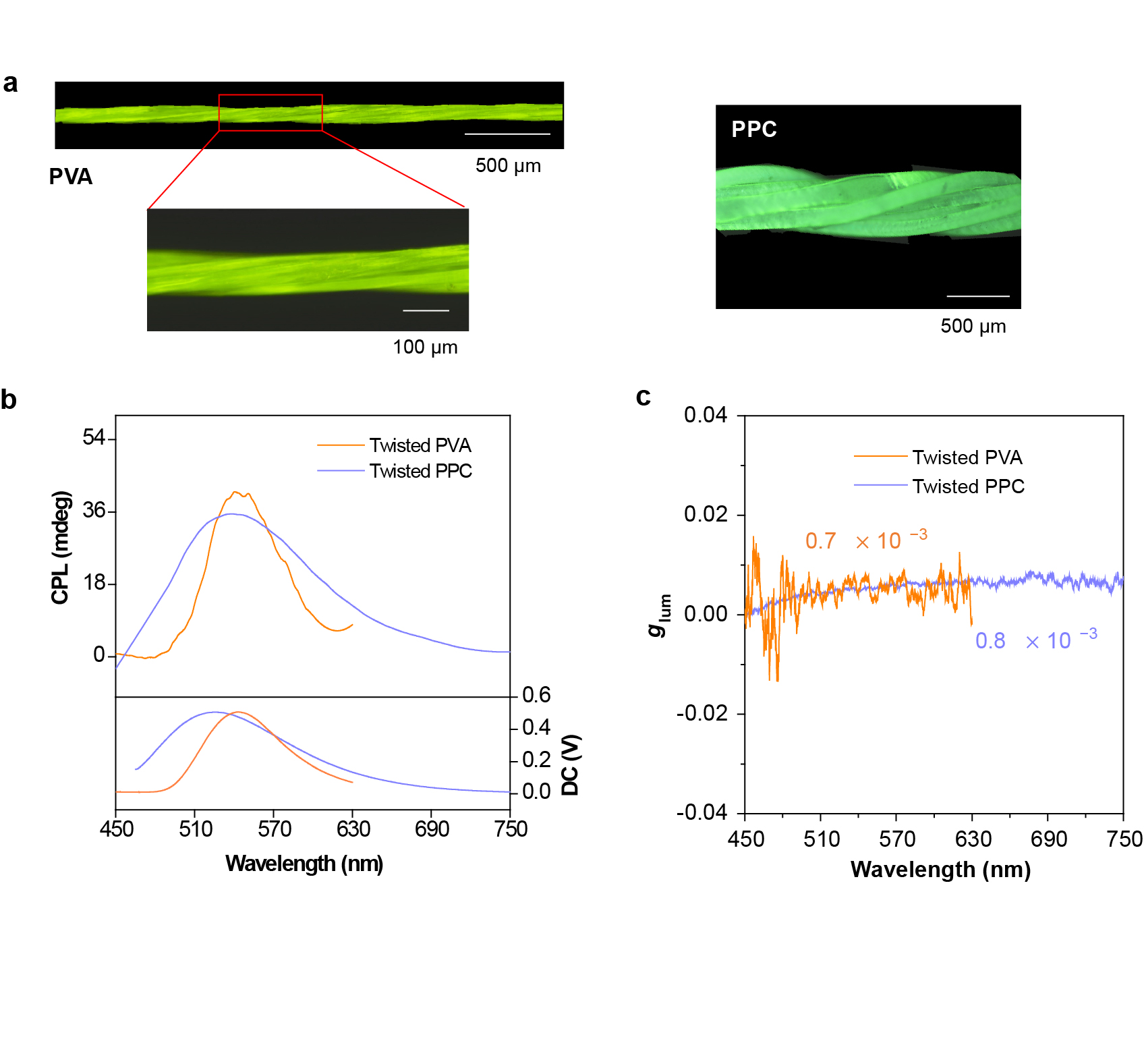
***

**Figure S22. Morphologies and CPL properties of twisted fibers with different polymer matrices.** a) Fluorescence micrographs of twisted PVA (left panel) and PPC (right panel) fiber, and b) their corresponding CPL spectra and c) *g*_lum_ spectra.

***2.12 CPL activity of commercially twisted fluorescent*** ***fibers***


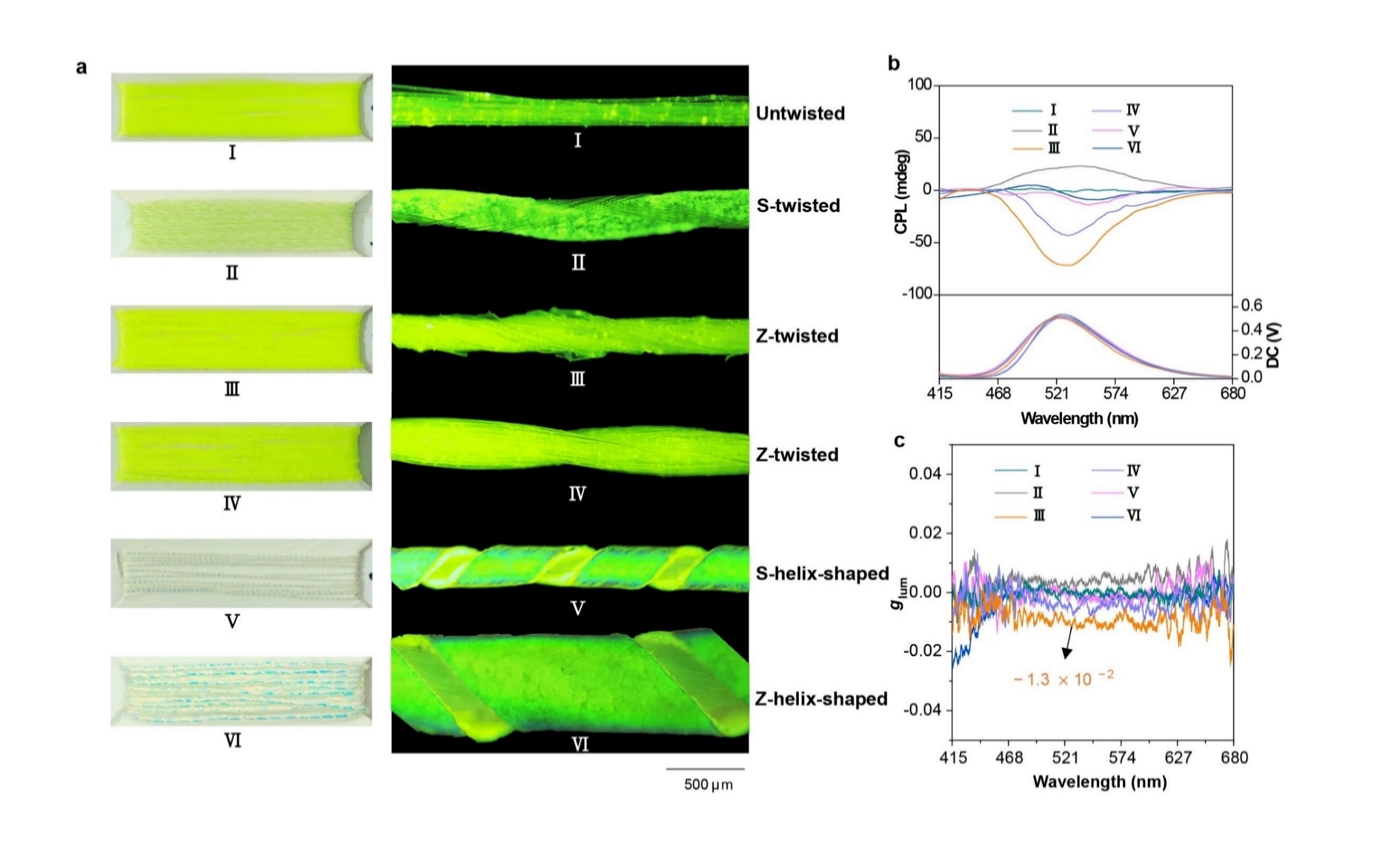


**Figure S23. Morphologies and CPL properties of the commercially twisted fluorescent threads.** a) Optical macrographs (left) and fluorescence micrographs (right) of commercially twisted fluorescent threads and b) their corresponding CPL spectra and c) *g*_lum_ spectra.

1. ***References***

[1] L. Zhou, L. Zheng, X. Yu, M. Gao, C. Xu, Y. Ge, T. Bai, J. Wen, Y. Cheng, M. Zhu, *Aggregate* **2023**, *4*, No. e338.

[2] Y. Cheng, J. Wang, Z. Qiu, X. Zheng, N. L. C. Leung, J. W. Y. Lam, B. Z. Tang, *Adv. Mater.* **2017**, *29*, 1703900.

[3] Z. Qiu, E. K. K. Chu, M. Jiang, C. Gui, N. Xie, W. Qin, P. Alam, R. T. K. Kwok, J. W. Y. Lam, B. Z. Tang, *Macromolecules* **2017**, *50*, 7620−7627.

[4] M. Khorloo, X. Yu, Y. Cheng, H. Zhang, S. Yu, J. W. Y. Lam, M. Zhu, B. Z. Tang, *ACS Nano* **2021**, *15*, 1397.
